# Accurate Bayesian segmentation of thalamic nuclei using diffusion MRI and an improved histological atlas

**DOI:** 10.1016/j.neuroimage.2023.120129

**Published:** 2023-07-01

**Authors:** Henry F.J. Tregidgo, Sonja Soskic, Juri Althonayan, Chiara Maffei, Koen Van Leemput, Polina Golland, Ricardo Insausti, Garikoitz Lerma-Usabiaga, César Caballero-Gaudes, Pedro M. Paz-Alonso, Anastasia Yendiki, Daniel C. Alexander, Martina Bocchetta, Jonathan D. Rohrer, Juan Eugenio Iglesias, for the Alzheimer's Disease Neuroimaging Initiative

**Affiliations:** a Centre for Medical Image Computing, Department of Medical Physics and Biomedical Engineering, University College London, UK; b Martinos Center for Biomedical Imaging, Massachusetts General Hospital and Harvard Medical School, USA; c Department of Health Technology, Technical University of Denmark, Denmark; d Computer Science and Artificial Intelligence Laboratory, Massachusetts Institute of Technology, USA; e Human Neuroanatomy Laboratory, University of Castilla-La Mancha, Spain; f BCBL. Basque Center on Cognition, Brain and Language, Spain; g Ikerbasque, Basque Foundation for Science, Bilbao, Spain; h Centre for Medical Image Computing, Department of Computer Science, University College London, UK; i Dementia Research Centre, Department of Neurodegenerative Disease, UCL Queen Square Institute of Neurology, University College London, UK; j Centre for Cognitive and Clinical Neuroscience, Department of Life Sciences, College of Health, Medicine and Life Sciences, Brunel University London, UK

**Keywords:** Thalamus, Atlasing, Diffusion MRI, Segmentation, Bayesian inference

## Abstract

The human thalamus is a highly connected brain structure, which is key for the control of numerous functions and is involved in several neurological disorders. Recently, neuroimaging studies have increasingly focused on the volume and connectivity of the specific nuclei comprising this structure, rather than looking at the thalamus as a whole. However, accurate identification of cytoarchitectonically designed histological nuclei on standard *in vivo* structural MRI is hampered by the lack of image contrast that can be used to distinguish nuclei from each other and from surrounding white matter tracts. While diffusion MRI may offer such contrast, it has lower resolution and lacks some boundaries visible in structural imaging. In this work, we present a Bayesian segmentation algorithm for the thalamus. This algorithm combines prior information from a probabilistic atlas with likelihood models for both structural and diffusion MRI, allowing segmentation of 25 thalamic labels per hemisphere informed by both modalities. We present an improved probabilistic atlas, incorporating thalamic nuclei identified from histology and 45 white matter tracts surrounding the thalamus identified in ultra-high gradient strength diffusion imaging. We present a family of likelihood models for diffusion tensor imaging, ensuring compatibility with the vast majority of neuroimaging datasets that include diffusion MRI data. The use of these diffusion likelihood models greatly improves identification of nuclear groups versus segmentation based solely on structural MRI. Dice comparison of 5 manually identifiable groups of nuclei to ground truth segmentations show improvements of up to 10 percentage points. Additionally, our chosen model shows a high degree of reliability, with median test-retest Dice scores above 0.85 for four out of five nuclei groups, whilst also offering improved detection of differential thalamic involvement in Alzheimer’s disease (AUROC 81.98%). The probabilistic atlas and segmentation tool will be made publicly available as part of the neuroimaging package FreeSurfer (https://freesurfer.net/fswiki/ThalamicNucleiDTI).

## Highlights

• We add diffusion MRI to Bayesian thalamic nuclei segmentation with structural MRI.
• Adding fiber tracts to probabilistic atlases enables orientation modelling.
• Thalamus segmentation from joint structural and diffusion MRI improves accuracy.
• Atlas and companion segmentation code are freely distributed with FreeSurfer.

## 1 Introduction

The thalamus has traditionally been considered a relay station for information in the brain, with extensive connections to both cortical and subcortical structures (Behrens, Johansen-Berg, Woolrich, Smith, Wheeler-Kingshott, et al., 2003, Schmahmann, 2003). As such, it integrates information processing between cortical regions (Hwang, Bertolero, Liu, D’Esposito, 2017, Sherman, 2007, Sherman, 2016) and is associated with a wide range of functions including cognition, memory, sensory and motor functions, regulation of consciousness and spoken language among others (Fama, Sullivan, 2015, Schmahmann, 2003, Sherman, Guillery, 2001). Additionally, neurodegenerative pathological processes in the thalamus have been associated with Alzheimer’s disease (*AD*) (de Jong, van der Hiele, Veer, Houwing, Westendorp, et al., 2008, Zarei, Patenaude, Damoiseaux, Morgese, Smith, et al., 2010), frontotemporal dementia (Bocchetta, Gordon, Cardoso, Modat, Ourselin, et al., 2018, McKenna, Li Hi Shing, Murad, Lope, Hardiman, et al., 2022), Huntington’s disease (Aron, Schlaghecken, Fletcher, Bullmore, Eimer, et al., 2003, Kassubek, Juengling, Ecker, Landwehrmeyer, 2005) and multiple sclerosis (Minagar, Barnett, Benedict, Pelletier, Pirko, et al., 2013, Planche, Su, Mournet, Saranathan, et al., 2019).

With such wide established connections and functions, the thalamus is a frequent target in MRI-based neuroimaging studies and a focus for research in relation to both healthy and disordered brain function. This creates a need for reliable identification of thalamic borders. Therefore, the thalamus is defined by several structural MRI (*sMRI*) segmentation methods, including multi-atlas segmentation (Heckemann et al., 2006), Bayesian segmentation (Puonti et al., 2016) and convolutional neural networks (*CNNs*) (Billot, Greve, Van Leemput, Fischl, Iglesias, et al., 2020, Henschel, Conjeti, Estrada, Diers, Fischl, et al., 2020, Wachinger, Reuter, Klein, 2018). Additionally, the thalamus has been included in popular image processing packages, including FreeSurfer’s (Fischl, 2012) recon-all stream, which uses a probabilistic atlas of anatomy and MRI intensity (Fischl et al., 2002), and the FMRIB Software Library (*FSL*) (Smith et al., 2004), which includes a model of shape and appearance in its implementation (FIRST) (Patenaude et al., 2011).

The methods above segment the thalamus as a single label, however in reality it is a complex and heterogeneous structure. It is composed of 14 major nuclei, which may be split further into 50 subnuclei depending on the level of detail in the classification and agreement on neuroanatomical definition. There are multiple such definitions with varying numbers of subnuclei (Jones, 2012, Mai, Majtanik, 2019, Morel, 2007). These nuclei have distinct patterns of connections with other brain regions and subserve different functions, including associative, sensory, motor, cognitive and limbic (Schmahmann, 2003). For example, the ventral lateral posterior nucleus is involved in motor function through connections with the cerebellum and the motor cortex, while the mediodorsal nucleus has connections with the prefrontal cortex and plays a role in cognitive and emotional processes (Mai, Forutan, 2012, Schmahmann, 2003). In addition, neuropathological studies have demonstrated preferential involvement of certain thalamic nuclei in several conditions, such as the caudal intralaminar nuclei in Parkinsons disease (Henderson et al., 2000), the anterior nuclei in AD (Braak, Braak, 1991, Braak, Braak, 1991), and the pulvinar in the *C9orf72* genetic subtype of frontotemporal dementia (Vatsavayai et al., 2016). These studies provide strong motivation for the design of automated segmentation algorithms that accurately define thalamic nuclei *in vivo*, enabling identification of reliable and precise biomarkers.

Different approaches have been used to segment thalamic nuclei. There are segmentation strategies that attempt to directly register histology derived labels to MRI. For instance, manually labelled histology can be used to generate a reference space atlas that may then be applied to *in vivo* MRI through registration-based segmentation (Jakab, Blanc, Berényi, Székely, 2012, Krauth, Blanc, Poveda, Jeanmonod, et al., 2010, Sadikot, Mallar Chakravarty, Bertrand, Rymar, et al., 2011). However, such approaches are limited by the difficulty in registering MR images with different contrasts. Other techniques define their label scheme based on information derived from the imaging data to be segmented. For example, diffusion MRI (*dMRI*) has been used to define thalamic regions by clustering voxels based on diffusion tensor imaging (*DTI*) indices (Mang et al., 2012) and orientation distribution functions (Battistella, Najdenovska, Maeder, Ghazaleh, Daducci, 2017, Semedo, Cardoso, Vos, Sudre, Bocchetta, et al., 2018). Other studies have divided the thalamus into regions based on their cortical connectivity, either through resting-state functional MRI time course correlations (Zhang et al., 2008) or dMRI tractography (Behrens, Johansen-Berg, Woolrich, Smith, Wheeler-Kingshott, et al., 2003, Johansen-Berg, Behrens, Sillery, Ciccarelli, Thompson, et al., 2005). However, exactly how thalamic regions defined by functional MRI relate to neurobiology is not fully understood (Eickhoff et al., 2015) and there is some indication that tractography-based segmentations are insensitive to the internal structure of the thalamus (Clayden et al., 2019).

The development of advanced MRI acquisitions has also allowed for atlases to be defined from manual segmentation of *in vivo* imaging directly, due to improved resolution and contrast. For example, guided by histological atlases, it has been possible to manually identify nuclei on advanced sMRI acquired at 7T (Liu, D’Haese, Newton, Dawant, 2020, Tourdias, Saranathan, Levesque, Su, Rutt, 2014) and on dMRI through short-track track density imaging (Basile et al., 2021). In particular, segmentations of 7T white-matter-nulled imaging have been used to generate both multi-atlas segmentation (”THOMAS” Su et al. 2019) and CNN (Umapathy et al., 2021) segmentation algorithms. However, these segmentations do not have the full level of detail present in histological atlases and performance is impacted by changes in acquired contrast, due to domain gap effects for CNNs and poorer registration in multi-atlas segmentation.

**Fig. 1:**
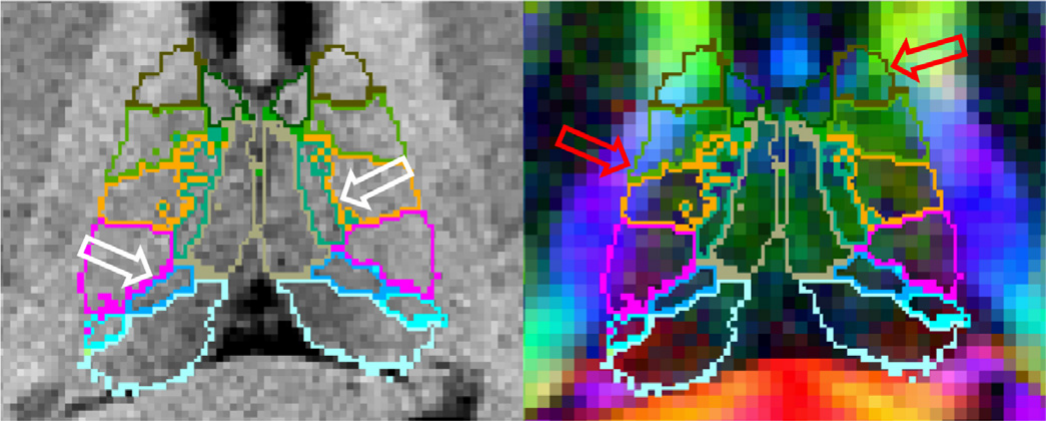
Thalamic segmentation of a T1-weighted structural MRI overlaid on the co-registered T1-weighted image (left) and a co-registered directionally encoded colour FA image (right). High contrast between medial and lateral thalamic regions on structural imaging improves the accuracy of these boundaries (white arrows). However, low contrast between the lateral thalamus and white matter causes over-segmentation into the internal capsule, which can easily be discerned in the colour FA image (red arrows). (For interpretation of the references to colour in this figure legend, the reader is referred to the web version of this article.)

Aiming to provide detailed segmentations of thalamic nuclei that is robust to changes in MRI acquisition and contrast, we previously constructed a probabilistic atlas of the thalamus and surrounding tissue from manually labelled histology (Iglesias et al., 2018). We then combined this atlas with Bayesian inference methods (Ashburner, Friston, 2005, Pohl, Fisher, Grimson, Kikinis, Wells, 2006, Van Leemput, Maes, Vandermeulen, Suetens, 1999, Wells, Grimson, Kikinis, Jolesz, 1996) to allow segmentation of 25 bilateral histological labels from sMRI. This approach had the advantage that the intensity model of each label was learned from the target image, reducing dependence of the resulting segmentations on the type of sMRI acquisition contrast. However, sMRI acquisitions can show poor contrast in some areas, leading to errors in segmentation that become apparent when overlaid on dMRI. For example, Fig. 1 shows that our previous method can accurately follow the boundary between groups of medial and lateral nuclei, but the lack of contrast between lateral nuclei and white matter can lead to oversegmentation into the internal capsule.

The availability of complementary information from dMRI sequences provides a possible avenue for minimising such segmentation errors. An increasing number of large multi-site neuroimaging studies, including the Human Connectome Project (*HCP*) (Van Essen et al., 2013), the Alzheimer’s Disease Neuroimaging Initiative (ADNI) (Jack et al., 2008), and the GENetic Frontotemporal dementia Initiative (GENFI) (Rohrer et al., 2015) are acquiring both structural and diffusion MRI. Additionally, use of DTI combined with registration-based segmentation has been proposed for segmentation of the whole thalamus in subjects where T1-weighted MRI contrast is very low (Al-Saady et al., 2022). As can be seen in Fig. 1, dMRI shows good contrast between the thalamus and the adjacent white matter, while structural MRI provides better contrast between the medial nuclei and cerebrospinal fluid (*CSF*) as well as higher resolution. Therefore, we look towards creating joint models of structural and diffusion MRI, incorporating likelihood models of DTI such as those used in the modelling of white matter fibres (Jian and Vemuri, 2007).

We present an extension of our structural Bayesian inference segmentation algorithm to incorporate dMRI. We focus on DTI due to the ease of fitting tensors to diffusion-weighted images, even from legacy data or in studies with short acquisitions. We explore our recently proposed diffusion likelihood model, combining the Dimroth-Scheidegger-Watson (*DSW*) and Beta distributions (Iglesias et al., 2019). We compare this model to both the Wishart distribution, from fibre modelling literature (Jian and Vemuri, 2007), and the log-Gaussian distribution, influenced by tensor interpolation methods (Arsigny et al., 2006). Additionally, we build on our previous histological atlas of the thalamus by adding 45 labels for white matter tracts passing adjacent to the thalamus, allowing the DTI likelihood models to capture the varying directionality of fibers in white matter without becoming sensitive to non-white-matter tissue. The resulting segmentation method allows constraints to be imposed independently on both the structural and diffusion modelling by including separate shared parameter models, enforcing reflective symmetry, incorporating prior distributions on likelihood parameters, and re-weighting likelihood terms to account for the lower resolution of DTI.

This paper is structured as follows. In Section 2 we outline our joint segmentation method. This includes explanations of: the general Bayesian inference model; the model fitting and segmentation process; the three likelihood models; the atlas and its construction; and general implementation details. In Section 3 we evaluate our joint segmentation method on both high and low resolution data. This evaluation includes: model optimisation and evaluation on a population template constructed from both T1-weighted MP-RAGE and DTI images; evaluation of the optimised models on subjects from HCP, providing comparison to manual ground truth and test-retest reliability; and test-retest and indirect evaluation on conventional quality data. Section 4 concludes the paper.

## 2 Bayesian segmentation of brain MRI

### 2.1 Probabilistic model and Bayesian inference

Here we outline the theory and implementation of our Bayesian segmentation algorithm. As in existing Bayesian segmentation literature (Ashburner, Friston, 2005, Iglesias, Augustinack, Nguyen, Player, Player, et al., 2015, Puonti, Iglesias, Van Leemput, 2016, Van Leemput, Maes, Vandermeulen, Suetens, 1999, Zhang, Brady, Smith, 2001), our strategy relies on modelling the voxel-wise data as observable random variables. These follow a different distribution for each label class in a supplied deformable probabilistic atlas of the volume encompassing the thalamus (Iglesias, Insausti, Lerma-Usabiaga, Bocchetta, Van Leemput, et al., 2018, Van Leemput, 2009). Both the voxel-data distributions and deformation of the atlas are parameterised by hidden random variables dependent on the subject and image acquisition. Estimating these hidden random variables allows us to generate a voxel-wise probability of membership in each label class (Ashburner, Friston, 2005, Van Leemput, Maes, Vandermeulen, Suetens, 1999). In the Bayesian approach, this is used to construct the posterior probability of a labelling (or segmentation) given paired sMRI and dMRI data.

For the purposes of this method we assume that both the sMRI and dMRI have been registered and resampled to the same grid comprised of voxels indexed by v∈{1,⋯,V}. We denote the labelling of these voxels by $L=[l_{1},\ldots ,l_{V}]$, with $l_{v}\in \left\{1,\ldots ,C\right\}$ – where C is the number of label classes in our model. Similarly, we construct a matrix $S=[s_{1},\ldots ,s_{V}]$ holding vectors of sMRI voxel data, $s_{v}$, and matrix $D=[d_{1},\ldots ,d_{V}]$ to hold the dMRI voxel data, $d_{v}$. We explore different representations of $d_{v}$ in later sections.

Using this notation and applying Bayes’ rule, the posterior probability of a specific labelling for a pair of sMRI and dMRI scans of a subject is:

(1) p(L|S,D)∝p(S,D|L)p(L),

and the labelling that maximises Eq.  (1) is known as the maximum a posteriori (*MAP*) estimate for the segmentation. To obtain this MAP estimate we need both the *likelihood* distribution, p(S,D|L), of our imaging data given a segmentation, and a *prior* distribution, p(L), generated from prior anatomical knowledge of the thalamus and its surroundings. As these can be used to generate random scans by sampling first from the prior then from the likelihood, segmentation can be thought of as fitting a generative probabilistic forward model to our data and “inverting” it to obtain the labelling.

**Fig. 2:**
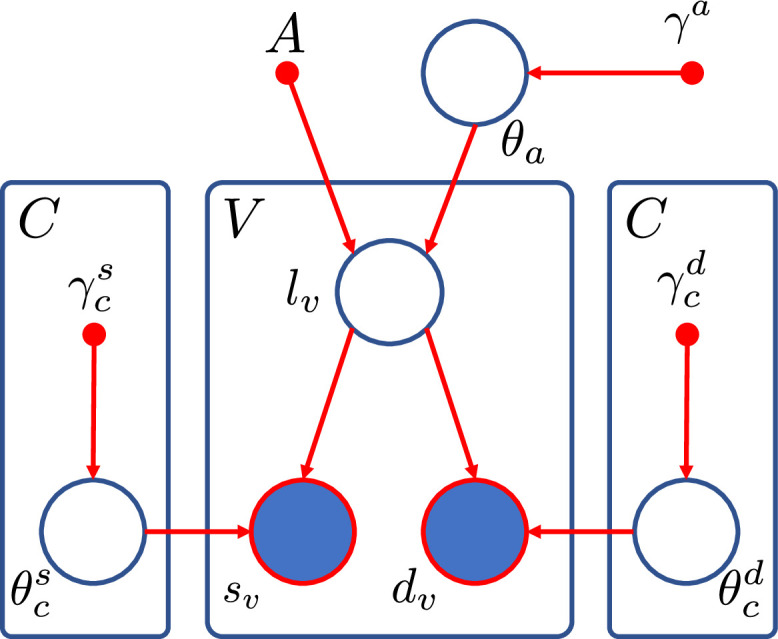
Graphical model of the proposed framework. Larger circles represent random variables with open circles for the hidden variables (θ,l), and shaded circles for the observed variables (s,d). Smaller solid circles are deterministic parameters such as the atlas (A) and encoded prior information (γ). Rectangles indicate replication across voxels (V) or classes (C).

To make the problem in Eq.  (1) tractable, we assume: *i)* that both the likelihood and prior factorise over voxels and *ii)* that the sMRI and dMRI are independent of each other given the labels. The exact graphical model of our framework is shown in Fig. 2. At the top of this model we define the prior distribution on the labels, beginning with a probabilistic atlas A. This atlas is constructed within a reference brain space, meaning it is likely to match the topology of any segmentation subject, but will require deformation to match accurately. The atlas A provides, at each spatial location, the prior probability of observing each neuroanatomical label class. We define A on a deformable tetrahedral mesh, where each vertex has an associated vector of class probabilities, and barycentric interpolation can be used to obtain probabilities at non-vertex locations (Van Leemput, 2009). We define a set of parameters, $\theta^{a},$ that move the mesh nodes to deform the atlas into the space of the target MRI voxel grid, accommodating the anatomical variability across subjects. These parameters are themselves a sample from a distribution that is regularised by setting the stiffness $\gamma^{a}$, preventing folding of the atlas mesh and preserving topology. We then assume that our labelling L is sampled from the categorical distribution over classes defined by the deformed atlas, with each voxel location sampled independently allowing factorisation.

Given L we can define the likelihood model for our observed data. We assume that the sMRI and dMRI are conditionally independent from each other and across voxels given the labelling, with $s_{v}$ and $d_{v}$ modelled as samples from separate distributions parameterised by $\theta_{c}^{s}$ and $\theta_{c}^{d}$ respectively. These hidden parameters are dependent on the corresponding label $l_{v}=c$. Any prior knowledge on these parameters is encoded in prior distributions controlled by hyperparameters $\gamma_{c}^{s}$ and $\gamma_{c}^{d}$.

Under these assumptions we can define the full joint probability density function (*PDF*) for Fig. 2 as

(2) $$\begin{array}{ccc} & & p(S,D,L,\theta |A,\gamma )\\ & & \;=p\left(S|L,\theta^{s}\right)p\left(D|L,\theta^{d}\right)p\left(L|A,\theta^{a}\right)p\left(\theta |\gamma \right)\\ & & \;=\left(\prod_{v=1}^{V}p\left(s_{v}|\theta_{l_{v}}^{s}\right)p\left(d_{v}|\theta_{l_{v}}^{d}\right)p\left(l_{v}|A,\theta^{a}\right)\right)\\ & & \;\;\;\;\;\left(\prod_{c=1}^{C}p\left(\theta_{c}^{s}|\gamma_{c}^{s}\right)p\left(\theta_{c}^{d}|\gamma_{c}^{d}\right)\right)p\left(\theta^{a}|\gamma^{a}\right), \end{array}$$

where $\theta =\{\theta_{c}^{s},\theta_{c}^{d},\theta_{c}^{a}\}$ and $\gamma =\{\gamma_{c}^{s},\gamma_{c}^{d},\gamma_{c}^{a}\}$.

With the model described by Fig. 2 and Eq.  (2) we can formulate the MAP estimate for our segmentation as

(3) $$L_{\mathrm{MAP}}=\underset{L}{arg max}p\left(S,D|L,A,\gamma \right)p\left(L|A,\gamma \right)=\underset{L}{arg max}\int p\left(S,D|L,\theta ,A\right)p\left(L|\theta ,A\right)p\left(\theta |S,D,A,\gamma \right)d\theta .$$

However, integrating the joint PDF over the full space of possible parameters θ is intractable. For this reason we make the standard assumption that the posterior distribution of the hidden parameters is heavily peaked around the mode, $p\left(\theta |S,D\right)≃\delta \left(\theta -\hat{\theta }\right)$. In this way, we can segment our images by applying Bayes’ rule to Eq.  (2) and marginalising over the hidden labelling L to obtain these optimal hidden parameters (so called ”point estimates”):

(4) $$\begin{array}{ccc} \hat{\theta } & = & \underset{\left\{\theta^{a},\theta^{s},\theta^{d}\right\}}{arg max}[p\left(\theta^{a}|\gamma^{a}\right)p\left(\theta^{s}|\gamma^{s}\right)p\left(\theta^{d}|\gamma^{d}\right)\\ & & \;\;\;\sum_{L}p\left(S,D|L,\theta^{s},\theta^{d}\right)p\left(L|\theta^{a},A\right)], \end{array}$$

and then optimising L to obtain the MAP estimate

(5) $$L_{\mathrm{MAP}}=\underset{L}{arg max}\;p\left(S,D|L,\hat{\theta },A\right)p\left(L|\hat{\theta },A\right).$$

### 2.2 Parameter estimation and segmentation

The first step is to estimate the optimal hidden parameters $\hat{\theta }$ from Eq.  (4). We begin by formulating the likelihood PDFs for both sMRI and dMRI as mixture models. Each label class in the atlas is described by its own mixture model constructed using a selection from G structural and W diffusion component distributions. The likelihoods of $s_{v}$ and $d_{v}$ given membership of voxel v in class c are then

(6) $$p\left(s_{v}|\theta_{c}^{s}\right)=\sum_{i}g_{c,i}p\left(s_{v}|\theta_{i}^{s}\right),\;p\left(d_{v}|\theta_{c}^{d}\right)=\sum_{j}w_{c,j}p\left(d_{v}|\theta_{j}^{d}\right).$$

Here, $g_{c,i}\geq 0$ and $w_{c,j}\geq 0$ are mixture weights in the model of label class c indicating the contribution of the i-th sMRI and j-th dMRI components to the appearance of the class in the respective modality. These distributions are parameterised by $\theta_{i}^{s}$ and $\theta_{j}^{d},$ respectively, with i∈1,…,G and j∈1,…,W. In both cases the sum over the component weights for a given class must be equal to one, $\sum_{i}g_{c,i}=1$ and $\sum_{j}w_{c,j}=1,$ ensuring all white- and grey-matter class boundaries are informed by both structural and diffusion contrast. This formulation provides a high degree of flexibility, allowing us to specify *a priori* combinations of classes that may be modelled using the same parameters.

Combining Eq. (6) with (2), (4) and (4) and taking logarithms we can then obtain an objective function to be optimised with respect to the distribution parameters,

(7) $$\begin{array}{ccc} & & O(\theta |S,D,A,\gamma )\\ & & \;=\log p\left(\theta^{a}|\gamma^{a}\right)+\sum_{i}^{G}\log p\left(\theta_{i}^{s}|\gamma_{i}^{s}\right)+\sum_{j}^{W}\log p\left(\theta_{j}^{d}|\gamma_{j}^{d}\right)\\ & & \;+\;\sum_{v}^{V}\log\sum_{c}^{C}p\left(l_{v}^{c}|A,\theta^{a}\right)\left[\sum_{i}^{G}g_{c,i}p\left(s_{v}|\theta_{i}^{s}\right)\right]\left[\sum_{j}^{W}w_{c,j}p\left(d_{v}|\theta_{j}^{d}\right)\right]. \end{array}$$

To optimise Eq.  (7) we adapt the approach proposed by Puonti et al. (2016). In this approach the atlas deformation parameters and likelihood parameters are optimised iteratively in a coordinate ascent scheme, with each being optimised while the other is fixed. The optimisation of the $\theta^{a}$ is performed using a standard conjugate gradient operator with the deformation prior $p(\theta^{a}|\gamma^{a})$ taking the form of the penalty term defined by Ashburner et al. (2000). The likelihood parameters $\theta^{s}$ and $\theta^{d}$ are then optimised using a Generalised Expectation Maximisation (*GEM*) algorithm (Dempster, Laird, Rubin, 1977, Van Leemput, Maes, Vandermeulen, Suetens, 1999), iterating between expectation (*E*) and Maximisation (*M*) steps.

*E step:* In the E step, we build a lower bound Q(θ) for the objective function in Eq.  (7) using Jensen’s inequality:

(8) $$\begin{array}{cc} Q(\theta ) & =\log p\left(\theta^{a}|\gamma^{a}\right)+\sum_{i}^{G}\log p\left(\theta_{i}^{s}|\gamma_{i}^{s}\right)+\sum_{j}^{W}\log p\left(\theta_{j}^{d}|\gamma_{j}^{d}\right)\\ & \;+\sum_{v,c,i,j}q_{v}^{c,i,j}\log\left[p\left(l_{v}^{c}|A,\theta^{a}\right)p\left(s_{v}|\theta_{i}^{s}\right)p\left(d_{v}|\theta_{j}^{d}\right)\right]\\ & \;-\sum_{v,c,i,j}q_{v}^{c,i,j}\left[\log q_{v}^{c,i,j}-\log g_{c,i}-\log w_{c,j}\right]. \end{array}$$

Here $l_{v}^{c}$ indicates the event that the voxel label $l_{v}=c$ and $q_{v}^{c,i,j}$ is a soft segmentation at the current parameter estimates indicating the combination of class c, sMRI distribution i and dMRI distribution j:

(9) $$q_{v}^{c,i,j}=\frac{g_{c,i}w_{c,j}p\left(l_{v}^{c}|A,\theta^{a}\right)p\left(s_{v}|\theta_{i}^{s}\right)p\left(d_{v}|\theta_{j}^{d}\right)}{\sum_{\left\{c,i,j\right\}}g_{c,i}w_{c,j}p\left(l_{v}^{c}|A,\theta^{a}\right)p\left(s_{v}|\theta_{i}^{s}\right)p\left(d_{v}|\theta_{j}^{d}\right)}.$$

*M step:* In the generalised M step we attempt to increase the bound Q(θ) in Eq.  (8). We note that the two sets of distribution parameters $\theta_{i}^{s}$ and $\theta_{j}^{d}$ can be optimised individually, as they make independent contributions to the bound:

(10) $$Q_{s}\left(\theta_{i}^{s}\right)=\log\;p\left(\theta_{i}^{s}|\gamma_{i}^{s}\right)+\sum_{v}^{V}\left[\sum_{c,j}q_{v}^{c,i,j}\right]\log p\left(s_{v}|\theta_{i}^{s}\right),$$

(11) $$Q_{d}\left(\theta_{j}^{d}\right)=\log p\left(\theta_{j}^{d}|\gamma_{j}^{d}\right)+\sum_{v}^{V}\left[\sum_{c,i}q_{v}^{c,i,j}\right]\log p\left(d_{v}|\theta_{j}^{d}\right).$$

These contributions can then be optimised using either closed form solutions or numerical methods, depending on the distribution used as we will describe in Section 2.3. Finally we can calculate the new optimal weightings as

(12) $$g_{c,i}=\frac{\sum_{\left\{v,j\right\}}q_{v}^{c,i,j}}{\sum_{\left\{v,i,j\right\}}q_{v}^{c,i,j}}\;w_{c,j}=\frac{\sum_{\left\{v,i\right\}}q_{v}^{c,i,j}}{\sum_{\left\{v,i,j\right\}}q_{v}^{c,i,j}}$$

*Segmentation:* The mesh deformation and likelihood parameter optimisation steps are repeated alternately until the objective function in Eq.  (7) has converged. At this point, we note that the formulation of the posterior factorises over voxels and the posterior probability of each class may be found by summing over the soft segmentations $q_{v}^{c,i,j}.$ Hence the final MAP estimate segmentation is given by

(13) $${\hat{l}}_{v}=\underset{c}{arg max}\sum_{i=1}^{G}\sum_{j=1}^{W}q_{v}^{c,i,j}.$$

### 2.3 Likelihoods

So far, we have outlined the Bayesian framework and segmentation process without specifying the likelihood models used for both sets of MRI data. The steps outlined above are not affected by the choice of distributions used. Here we provide an overview of the distributions used to model the sMRI and dMRI data, including the likelihood term and, where applicable, the prior over its parameters. Detailed equations for the calculation of PDF values as well as the optimisation of model parameters, θ, may be found in Section S.1 of the supplement.

#### 2.3.1 Structural MRI model

To model the sMRI intensities, we follow the Bayesian brain MR segmentation literature and use a mixture of Gaussian intensity distributions (Ashburner, Friston, 2005, Van Leemput, Maes, Vandermeulen, Suetens, 1999, Zhang, Brady, Smith, 2001). In this model the intensity values for each structural modality are held in the vector $s_{v}$ and the model parameters $\theta_{i}^{s}$ are the mean and covariance, $\{\mu_{i},\Sigma_{i}\},$ of the structural mixture component i. We choose to use the natural conjugate prior, the Normal-Inverse-Wishart distribution, on these Gaussian parameters. The likelihood and prior distributions are therefore

(14) $$p\left(s_{v}|\theta_{i}^{s}\right)∼N\left(\mu_{i},\Sigma_{i}\right),\;p\left(\mu_{i},\Sigma_{i}|\gamma_{i}^{s}\right)∼\mathrm{NIW}\left(M_{i}^{s},n_{i}^{s},\Psi_{i}^{s},\nu_{i}^{s}\right),$$

where $M_{i}^{s},n_{i}^{s},\Psi_{i}^{s}$ and $\nu_{i}^{s}$ encode any prior knowledge we may have on the structural distribution. Formulations for the structural PDFs and closed form solutions to the parameter M step parameter optimisations can be found in Section S.1.1 of the supplement.

#### 2.3.2 Diffusion MRI models

To model the dMRI data, we consider distributions over tensors estimated with DTI. Even though higher-order models can be used with modern dMRI acquisitions, using DTI models ensures that our method is compatible with virtually every dMRI dataset, including huge amounts of legacy data. In this work, we compare two competing models, based on the Wishart and Gaussian distributions, to our previously-proposed DSW-beta distribution (Iglesias et al., 2019).

*Wishart:* Following existing white matter fibre modelling literature, we look to the Wishart distribution (Jian and Vemuri, 2007). DTI produces at each voxel a covariance matrix describing the displacements of water molecules in the voxel. Therefore, the natural conjugate prior for these tensors is an Inverse-Wishart distribution. We use this in combination with a Gamma distribution on the degrees of freedom parameter (Görür and Rasmussen, 2010), with the effect of lowering the degrees of freedom and increasing the breadth of the resulting Wishart distributions. In this model, we define $d_{v}$ as the inverse of the diffusion tensor $T_{v}$. We then use the Wishart and Gamma distributions to model $d_{v}$ and $\theta_{j}^{d}$:

(15) $$d_{v}∼W\left(n_{j}^{d},V_{j}^{d}\right),\;\left(n_{j}^{d}-2\right)/2∼\Gamma \left(\alpha ,\beta \right),$$

where α and β are set to 0.5 and 1.5 respectively to provide a non-informative prior. Formulations for the Wishart PDFs and the optimisation problem in the M step can be found in Section S.1.2 of the supplement.

*Log-Gaussian:* This model is motivated by literature on the interpolation of DTI volumes. Direct interpolation of DTI can lead to swelling of the ellipsoids representing the diffusion tensors, but interpolating in the log domain reduces this effect (Arsigny, Fillard, Pennec, Ayache, 2006, Dryden, Koloydenko, Zhou, 2009). For this reason, and noting that the DTI tensors, $T_{v}$, are symmetric with only six independent variables, we define $d_{v}$ as a vector

(16) $$d_{v}=P\mathrm{vec}\left(\log T_{v}\right),\;\mathrm{vec}\left(\log T_{v}\right)=P^{⊤}d_{v},$$

where P is a constant 6×9 matrix (values listed in supplement) designed with the constraint that

(17) $$∥\log\left(T_{1}\right)-\log\left(T_{2}\right){∥}_{F}^{2}={∥d_{1}-d_{2}∥}_{2}^{2},$$

and therefore interpolation of the vectors $d_{v}$ is equivalent to interpolation of the tensors in the log domain. In this formulation the natural distribution to choose based on the distance metric in Eq.  (17) is a Gaussian distribution with a scalar variance

(18) $$d_{v}∼N\left(m_{j}^{d},\sigma_{j}^{d}\right).$$

We then define uniform priors on both $m_{j}^{d}$ and $\sigma_{j}^{d}$ due to the difficulty in informing these parameters a priori. Formulations for the log-Gaussian PDFs and the optimisation problem in the M step can be found in Section S.1.3 of the supplement.

*DSW-beta:* This model is a custom combination of two distributions proposed in our prior work (Iglesias et al., 2019). This was motivated by a desire to lower the dimensionality of $d_{v}$, leading to a reduction in extreme values of the likelihood that may overwhelm the prior. Here only the fractional anisotropy (*FA*), $f_{v}$, and the principal eigenvector, $\phi_{v}$, of the tensor $T_{v}$ are modelled so that $d_{v}=\left\{f_{v},\phi_{v}\right\}$. In this approach, we use the two parameter Beta distribution to model the FA as it is able to model both the location and dispersion of signals in the range [0,1]. We then use the DSW distribution to model $\phi_{v}$.

The DSW distribution is defined on the unit sphere and parameterised by a mean direction ψ and a concentration κ, giving a PDF of the form

(19) $$p\left(\phi |\psi ,\kappa \right)={\left[Z(\kappa )\right]}^{-1}\exp\left\{\kappa {\left({\left(\psi \right)}^{⊤}\phi \right)}^{2}\right\},$$

where Z(κ) is a normalising constant given by the Kummer function in 3D (Mardia et al., 2000). As the DSW distribution is antipodally symmetric, it accommodates the directional invariance of dMRI (Zhang et al., 2012). It is also rotationally symmetric around a mean direction and its opposite {ψ,−ψ:∥ψ∥=1}, with a dispersion around the mean parameterised by the concentration κ. This κ allows us to incorporate the higher directional dispersion in voxels with lower FA by multiplying the component specific concentration by the voxel FA to give an effective concentration for each voxel. The likelihood distribution in this formulation of the dMRI is therefore a joint DSW-beta distribution

(20) $$f_{v}∼B\left(\alpha_{j}^{d},\beta_{j}^{d}\right),\;\phi_{v}∼\mathrm{DSW}\left(\psi_{j}^{d}|f\kappa_{j}^{d}\right).$$

Formulations for the DSW-beta PDFs and M step can be found in Section S.1.4 of the supplement.

### 2.4 Prior distribution: An improved probabilistic atlas of the thalamus

**Fig. 3:**
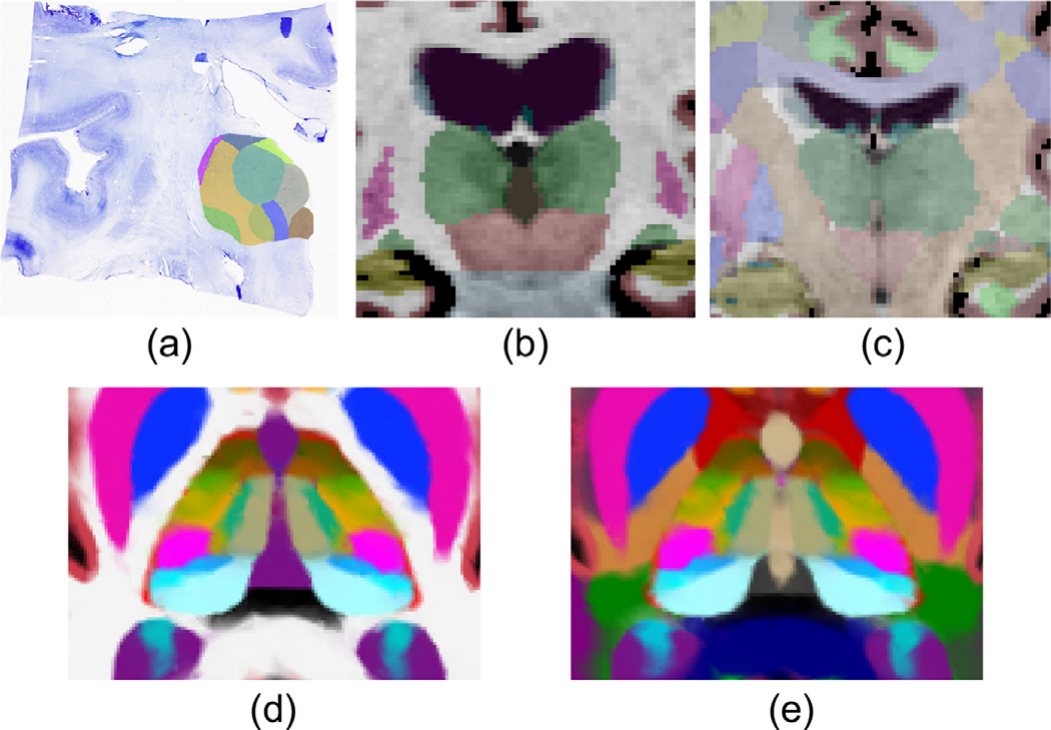
(a-c) Types of segmentations used to build the atlas. (a) Coronal histological section of the thalamus, with manual delineations of the nuclei. (b) Coronal slice of an *in vivo* T1-weighted MRI scan, with manual delineations for whole brain structures. (c) Similar coronal slice of one of the new 16 cases, with the white matter subdivided into tracts. (d-e) Corresponding axial slices of the previous and updated probabilistic atlases; colours are linear combinations of look up table colours weighted by their corresponding probability in each version of the atlas. The original atlas (d) was trained with segmentations like the ones in (a-b), while the new atlas used (a-c).

In Iglesias et al. (2018), we presented a highly detailed probabilistic atlas of the human thalamus built from a combination of *in vivo* MRI and histology. The spatial distribution of the thalamic nuclei was learnt from manual delineations drawn on 3D reconstructed histological sections from 12 specimens (Fig. 3a), whereas 39 MRI scans with manual delineations (Fischl et al., 2002) were used to learn the distribution of surrounding tissue (Fig. 3b). Direct use of this atlas (Fig. 3d) in our new framework is not ideal, as the cerebral white matter was modelled using only two classes – one per hemisphere. While such a parsimonious model with a single component is adequate for modelling the unimodal distribution of white mater intensities in sMRI, it is largely insufficient to model the dMRI orientations. The distribution over white matter voxels is highly multimodal due to the variety of fibre tracts that traverse this tissue in different orientations.

In principle we could model such a complex distribution using a mixture model with many components. However, such an approach is likely to fail, as some of these components may end up modelling non-white-matter tissue. Instead, we have refined our atlas by subdividing the white matter surrounding the thalamus into 45 tracts. To achieve this, we complemented the training data in Iglesias et al. (2018) (12 *ex vivo* thalami and 39 *in vivo* whole brains) with *in vivo* sMRI/dMRI data from 16 additional subjects, that were labelled manually as part of an update (Maffei et al., 2021) to the TRACULA (TRacts Constrained by UnderLying Anatomy) package distributed with FreeSurfer (Yendiki et al., 2011).

The TRACULA training set (16 healthy adults from the publicly available MGH-USC HCP; Fan et al. 2016) consisted of dMRI data, acquired using 512 directions at a maximum b-value of 10,000 $s/mm^{2}$ with 1.5 mm isotropic spatial resolution, and sMRI T1-weighted data, acquired with an MPRAGE sequence at 1 mm isotropic resolution. Cortical parcellations and subcortical segmentations, including the whole thalami and cerebral white matter (left and right), were obtained through FreeSurfer (Dale, Fischl, Sereno, 1999, Fischl, van der Kouwe, Destrieux, Halgren, Ségonne, et al., 2004, Fischl, Salat, Busa, Albert, Dieterich, et al., 2002, Fischl, Sereno, Dale, 1999). Whole-brain probabilistic tractograms were generated for each subject using constrained spherical deconvolution approaches (Jeurissen, Tournier, Dhollander, Connelly, Sijbers, 2014, Tax, Jeurissen, Vos, Viergever, et al., 2014) and streamlines used to manually label 42 white matter tracts through a combination of inclusion and exclusion criteria (Maffei et al., 2021). Resulting tractograms were transformed to the sMRI of the subject using a boundary-based, affine registration method (Greve and Fischl, 2009) and converted into visitation maps. These soft segmentations were spatially smoothed with a Gaussian kernel (σ = 2mm). For each white matter voxel in the FreeSurfer subcortical segmentation, we replaced its label by the tract with the highest probability (unless such probability was below 5%), dividing the white matter into 42 tracts and a generic white matter class (Fig. 3c).

The three types of segmentations (Fig. 3a-c) were used to rebuild the atlas, using a technique that enables combining labellings with different levels of detail (Iglesias et al., 2015). As a last adjustment, we manually excluded tracts not passing adjacent to the thalamus and subdivided labels corresponding to regions with identified heterogeneity of dMRI contrast. This subdivision principally affected the anterior commissure and the tracts comprising the corpus callosum, which were split into their left and right hemisphere components to account for reflective symmetry. The resulting atlas therefore contains 45 final labels for the white matter tracts. Each of these subclasses can be modelled either with unimodal distributions or mixtures with very few components, effectively preventing the modelling of non-white-matter tissue. Additionally, the medial pulvinar nuclei (*PuM*) were also split into lateral and medial classes to account for the typically more left-right directionality of their lateral portion. This is consistent with known connectivity differences between the medial and lateral portions of the PuM (Benarroch, 2015). As this split in our atlas was not derived directly from histological labels, we model these two PuM classes separately during optimisation and merge them for output.

Fig. 3 shows a comparison of the new (Fig. 3e) and old (Fig. 3d) atlases. The voxel colours in Fig. 3(d-e) are a linear combination of the label colours, weighted by their corresponding probability in each version of the atlas, providing a visual representation of smooth changes in the atlas for regions at the boundary of multiple labels. The new atlas is almost identical to the original, with the addition of more specific labels in the white mater and PuM. However, as with our previous atlas, the reticular and other classes outside the thalamus are used only for modelling purposes and are merged into the background for output, resulting in the segmentation of 50 labels.

### 2.5 Implementation details

#### 2.5.1 Data preparation

We assume that the sMRI has been processed with FreeSurfer, which yields a bias field corrected image and a whole brain segmentation (*aseg.mgz*, Fischl et al. 2002). The labels in aseg.mgz are used to initialise both the atlas deformation (Iglesias, Augustinack, Nguyen, Player, Player, et al., 2015, Iglesias, Insausti, Lerma-Usabiaga, Bocchetta, Van Leemput, et al., 2018) and hyperparameters in the structural prior in Eq.  (14). In practice the hypermean $M_{i}^{s}$ is estimated from the median of the relevant label in this initial coarse segmentation, and $n_{i}^{s}$ relates to the number of voxels used in estimating $M_{i}^{s}$. However, it is more difficult to robustly inform prior distributions of the covariance, so we set both $\Psi_{i}^{s}$ and $\nu_{i}^{s}$ to zero to provide a non-informative prior, giving the set of prior parameters $\gamma_{i}^{s}=\;\left\{M_{i}^{s},n_{i}^{s}\right\}$.

We also assume that the source dMRI has been put through the preprocessing stages of TRACULA (Maffei, Lee, Planich, Ramprasad, Ravi, et al., 2021, Yendiki, Panneck, Srinivasan, Stevens, Zöllei, et al., 2011). This includes FSL’s eddy current and subject motion correction (Andersson and Sotiropoulos, 2016) before fitting the tensor model. Additionally, we identify DTI voxels with poor fits as those with tensors that have negative eigenvalues or FA outside the range [0,1]. These are replaced by a local average tensor constructed by convolution of the log space tensors with a 3D Gaussian kernel. These cleaned tensors are converted to the log domain (Arsigny et al., 2006) before interpolation to the voxel grid of the sMRI.

#### 2.5.2 Mixture model specification

The assignment of component distributions to label classes is one of the modelling choices that must be made before segmentation. We assign structural and diffusion components independently for each label class, defining what we will call the structural mixture model (*sMM*) and diffusion mixture model (*dMM*) respectively. In practice, this constrains most weights $g_{c,i}$ and $w_{c,j}$ to 0 or 1, with a single component distribution often shared between groups of labels. However, we do allow for many-to-many relationships between the label-classes and components. For example, allowing the structural appearance of the CSF label to be modelled by two Gaussian components, one for ”clean” CSF that is also used to model ventricle labels and one for ”messy” CSF that is shared with the choroid plexus.

For class likelihoods composed of multiple distributions, the non-zero weights are set to be equal for the first E step and initial component parameters are obtained by use of k-means clustering. Details of this clustering for each likelihood formulation can be found in Section S.3 of the supplement, while optimisation of the default sMM and dMM definitions is performed in Section 3.2.

#### 2.5.3 Reflective symmetry

A common regularising constraint applied in structural Bayesian segmentation algorithms is to use a single distribution to model structures present in both hemispheres, even if they are subsequently given separate labels denoting their hemisphere. Such constraints have a similar effect to increasing the sample size in fitting the distribution, reducing the effect of outliers from initialisation errors or local intensity variations on the resulting segmentation. Hence we enforce this constraint in our sMRI modelling as well as a similar constraint on the dMRI models.

Due to the directionality of dMRI data we cannot enforce a single distribution to model two contralateral structures as in sMRI. Instead we make the assumption that there is a reflectional symmetry between the distributions on either side of the midline. This can be visualised as reflecting the average ellipsoids described by each distribution in some plane such as the medial plane. However, in practice such a plane of reflective symmetry is unlikely to be aligned perfectly with the scanner coordinate system. For this reason we obtain the plane of reflection from the dMRI data itself, optimising a vector normal to the plane of reflection, r, initially assumed to be parallel to the left-right axis of the voxel grid.

Prior to each M step, we substitute reflected distribution parameters to the bound in Eq.  (8) and formulate the contribution of r, producing an optimisation that can be written in the form

(21) $$r=\underset{r:∥r∥=1}{arg max}\sum_{j=1}^{W}f\left(\theta_{j}^{d}\right)\sum_{v=1}^{V}h\left(q_{v}^{j},\theta_{j}^{d},d_{v},r\right).$$

Here, h(·) is a function of the location statistics (e.g. mean vectors for log-Gaussian and DSW-beta), the dMRI voxel data and the diffusion component posteriors, ensuring contributions to the objective are weighted by their certainty. Similarly, f(·) is a function of the dispersion statistics (e.g. precision, concentration or degrees of freedom) which ensures the contributions of each component distribution are weighted more strongly when more heavily peaked.

Detailed formulations for the reflection optimisation and joint distribution fitting can be found in Section S.1 of the supplement. In each case the objective is a fourth order polynomial in r with closed form first and second derivatives and can be optimised using an interior-point method. We can then jointly fit parameters for corresponding component distributions in the left and right hemispheres.

#### 2.5.4 Likelihood adjustment

Our model assumes that the resolutions of the dMRI and sMRI are identical. While datasets such as the HCP deviate from this assumption to a lesser degree, conventional quality datasets have much lower resolution for the dMRI in particular, for example T1-weighted images are typically acquired with each voxel dimension at approximately 1 mm while dMRI voxel dimensions can approach 2.5 mm in each direction. As we resample to the resolution of the sMRI, more dMRI voxels are used in likelihood parameter estimation than are available from the source imaging, which leads to overfitting of the dMRI. In practice, we counteract this effect by raising the contribution of the dMRI likelihood in Eq.  (7) to a fractional power ϵ, thereby downplaying the weight of the dMRI voxels in the objective.

To choose the value of ϵ we then examine the effect of this change on the M step bound in Eq.  (11), which becomes

(22) $$Q_{d}\left(\theta_{j}^{d}\right)=\log p\left(\theta_{j}^{d}|\gamma_{j}^{d}\right)+\epsilon \sum_{v}^{V}\left[\sum_{c,i}q_{v}^{c,i,j}\right]\log p\left(d_{v}|\theta_{j}^{d}\right).$$

Here we see that optimisation of the diffusion parameters is performed with contributions from a total of V voxels which have been obtained by interpolation from a smaller number of voxels $V^{d}$. By setting ϵ equal to the ratio of voxel sizes between dMRI and sMRI, the sum in Eq.  (22) becomes approximately equal to the sum over $V^{d}$ voxels, where the contribution of each source voxel is a weighted mean of the surrounding interpolated voxel contributions. Further details can be found in Section S.2 of the supplement.

## 3 Experiments and results

To quantitatively evaluate our method and compare between the three likelihood formulations we performed experiments using co-registered sMRI and dMRI from three datasets. In Section 3.1 we generate a population template from HCP subjects, and use it to identify manually segmentable labels corresponding to groups of labels from our histological atlas. In Section 3.2 we use this template to tune our method in a process of model selection. In Section 3.3 we evaluate application of our method to high resolution dMRI on subjects from HCP, including comparisons to manual segmentations and test-retest reliability. Finally, in Section 3.4 we evaluate application of our method to conventional quality dMRI. This includes test-retest reliability on images acquired locally at the University College London Dementia Research Centre (*UCL DRC*) and indirect evaluation on subjects with underlying pathologies by testing our method’s ability to distinguish between healthy controls and subjects with AD from the ADNI dataset.

In the following experiments, when comparing regions of interest (ROIs) corresponding to the same label in two separate segmentations we use the Dice Similarity Coefficient (*DSC*) and 95^th^ percentile of Hausdorff distance (*95HD*). For two ROIs X and Y these are defined as

(23) $$\mathrm{DSC}\left(X,Y\right)=\frac{2∥X\cap Y∥}{∥X∥+∥Y∥},$$

(24) $$95\text{HD}\left(X,Y\right)=\max(d_{95}\left(X,Y\right),d_{95}\left(Y,X\right)),$$

where ∥·∥ indicates the volume of the ROI and $d_{95}\left(X,Y\right)$ is the 95^th^ percentile of the set of distances between points on the ROI boundaries, $\{d_{x}=\min_{y\in S_{Y}}{\left|x-y|\right\}}_{x\in S_{X}}$

Additionally, when comparing to segmentations performed using our previous structural-only method (Iglesias et al., 2018), we show results produced using the code distributed as part of FreeSurfer 7.2. However, in an attempt to ensure fair quantitative comparisons, the default mesh stiffness parameter of this structural implementation was increased to match the joint model that had been developed on the HCP dataset. This improved both the DSC and 95HD structural results compared to the default FreeSurfer distribution. Visual comparisons of these structural segmentations with and without mesh stiffness tuning can be found in section S.6 of the supplement.

### 3.1 Population template and manual labels

When evaluating segmentation methods for medical images, it is common practice to compare the resulting label maps to a gold standard, usually obtained from manual delineation by a trained rater. However, manual delineation of 50 histological labels on *in vivo* MRI is infeasible, as many of the boundaries between are invisible at ∼1mm resolution. Manual segmentation protocols for larger groups of thalamic regions (with fewer labels) exist in the literature (Tourdias et al., 2014), but their anatomical definitions are incompatible with those of our histological labels, introducing bias and preventing direct and fair comparison. In this study, our goal is to compare the performance of our tool with a gold standard that is based on our 50 histological labels and informed by both sMRI and dMRI contrast. For this reason, we adapted these labels to define our own manual segmentation criteria for thalamic labels that can be accurately visualised and segmented on a combination of T1-weighted MPRAGE and directionally-encoded colour FA (*DEC-FA*); when labels of smaller thalamic nuclei were not identifiable from the intensity and contrast of the MRIs, these labels were combined and grouped together, so that the boundaries of the original 50 histological atlas labels can be easily matched and compared.

**Fig. 4:**
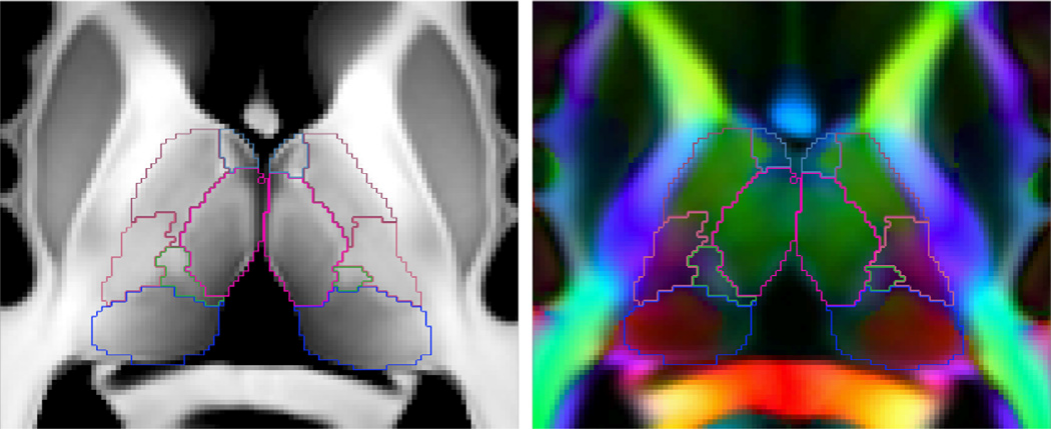
Representative axial view of the 10 label manual segmentation overlaid on the T1-weighted (left) and DEC-FA (right) population templates of the thalamus. Additional views are shown in Section S.8 of the supplement. Manually segmented label colour maps are given in Table 1 as are groupings for quantitative analysis.

The first step in defining these criteria was to create a high resolution template using 500 subjects from the WashU-UMN HCP dataset (Van Essen et al., 2013) and an unbiased template construction method (Joshi et al., 2004). We used three channels in the registration: T1-weighted intensity, T2-weighted intensity, and FA. In order to include directional information in the template, we used the final set of registrations to align and average the DTI tensors in the log domain. The resolution of the template is equal to the resolution of the HCP sMRI data, i.e., 0.7mm isotropic. Slices from the template are shown in Fig. 4.

**Table 1:** Summary of the label merging operations used to generate the manually segmented labels from histological atlas nuclei, and groupings of manual labels used for evaluation. Displayed colours follow the convention used in figures throughout this manuscript. Abbreviation definitions are listed in Section S.4 of the supplement. Visual comparisons of these three protocols are shown in Section S.8 of the supplement.

As a second step to define the gold standard for comparison, we registered the histological atlas to the template, producing a preliminary segmentation of 50 separate thalamic labels. This preliminary segmentation was then manually refined by an anatomy expert (JA, assisted by MB), to correct any anatomical errors from registration, and to combine those thalamic regions which were not reliably identifiable from the multi-modal template into labels which represent larger thalamic groups. This resulted in a set of 10 bilateral labels that were manually identifiable from the template.

The labeled template is used in Section 3.2 to aid in tuning our method. Additionally, features identified from this template segmentation are used as criteria in Section 3.3 to manually generate gold standard segmentations for comparison. These subject segmentations are performed without the aid of an automated preliminary segmentation. However, on application to individual HCP subjects, the reduced contrast and resolution resulted in increased ambiguity for some boundaries. Therefore we further combine the set of 10 manual *in vivo* labels generated for each subject into a final set of 5 coarser groupings, enabling evaluation without biasing results. Manual labels for the template can be seen in Fig. 4 and the correspondences between the evaluation groupings, manually segmented labels and original histological atlas labels can be seen in Table 1, with the exception of the Reticular, which is grouped with white matter as in our previous work (Iglesias et al., 2018).

### 3.2 Model selection

Practical implementation of the proposed framework requires decisions on how to share the sMM and dMM parameters (Section 2.5.2), which amounts to a model selection problem. In principle, our generative models enables the computation of the so-called model evidence, which enables comparison of models with different number of parameters. While theoretically appealing, computing this evidence requires marginalisation over all parameters, which leads to intractable integrals that require approximations. Instead, we selected the sMM/dMM groupings with a combination of prior knowledge and a systematic approach called “Technique for Order Preference by Similarity to Ideal Solution” (TOPSIS), which is a standard technique in operations research (Behzadian, Khanmohammadi Otaghsara, Yazdani, Ignatius, 2012, Hwang, Yoon, 1981).

*Structural groupings.* In our previous work, we used two Gaussian components to model the contrast difference between medial and lateral classes (Iglesias et al., 2018). Here, we added a third Gaussian modelling the medial portion of the PuM, which has a structural appearance closer to grey matter compared with the lateral portion of the PuM. We then compared the atlas prior and histograms of the template volumes to identify 33 possible sMMs grouping nuclei into three component distributions, which were considered by TOPSIS (detailed below).

*Diffusion groupings.* In Section 3.1 we defined 10 labels for each thalamus that are manually identifiable from combined sMRI and DEC-FA. However, inspection of the dMRI tensors within these regions found greater heterogeneity in some regions than in others. As additional borders within these labels could not be confidently matched with boundaries in the histological atlas, we examined multiple options for combining histological nuclei into larger structures to be fit with a component distribution. Including these additional boundaries, and allowing for the possibility of bimodal histograms for some labels, we arrived at 21 possible dMMs, grouping nuclei into between 11 and 13 component distributions.

*TOPSIS.* To optimise the choice of sMMs and dMMs in a systematic fashion, we tested each possible combination of sMM and dMM parameter groupings on the population template. We calculated Dice scores and 95HD for the whole thalamus as well as the ”grouping” and ”manual label” regions listed in Table 1. These Dice scores and distances were then used as measurement channels in the calculation of a single, normalised fitness score for each combination of shared parameters using TOPSIS.

**Fig. 5:**
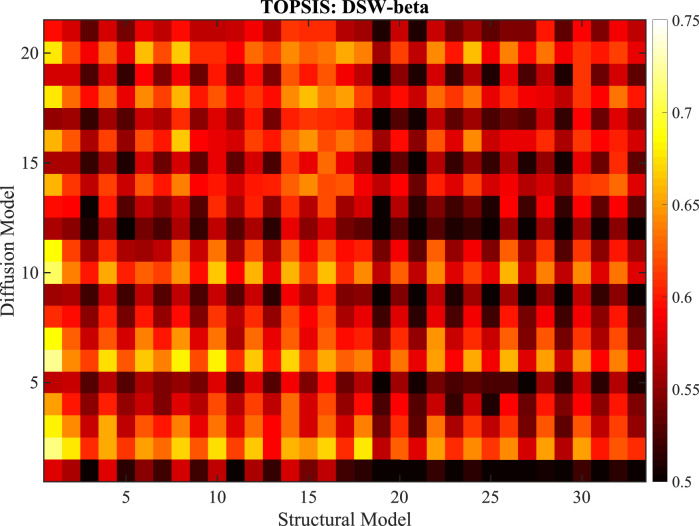
TOPSIS fitness plot for combinations of structural (horizontal axis) and diffusion (vertical axis) grouping models in the DSW-beta likelihood framework. Higher values indicate the combination of models produced Dice scores and boundary distances closer on average to the best value for each label. A mapping from model numbers to parameter groupings is provided as a spreadsheet in the supplementary material.

TOPSIS operates by first setting vectors of positive and negative ideal solutions for each measurement channel. For example, the positive ideal would be a vector containing the maximum Dice score achieved in each label as well as the minimum 95HD across all experiments. Each channel is then normalised and the $L^{2}$-norm distance is calculated giving scalar distance measures for each experiment from both the positive and negative ideal. These distances are then combined into a single similarity measure between 0 and 1 for each experiment, with 0 indicating the candidate achieves the worst performance in every Dice and 95HD measurement and 1 indicating the candidate achieves the best in each. These scores provide an effective fitness measure balancing the desire to achieve high precision measures of differing types for multiple labels while penalising poor performance in other measurements. The resulting scores for the DSW-beta model is shown in Fig. 5; equivalent plots for the Wishart and DSW-beta models may be found in section S.5 of the supplement. The chosen models are provided in a spreadsheet in the supplementary material as well as descriptions of all candidate models.

### 3.3 Application to high resolution dMRI

**Fig. 6:**
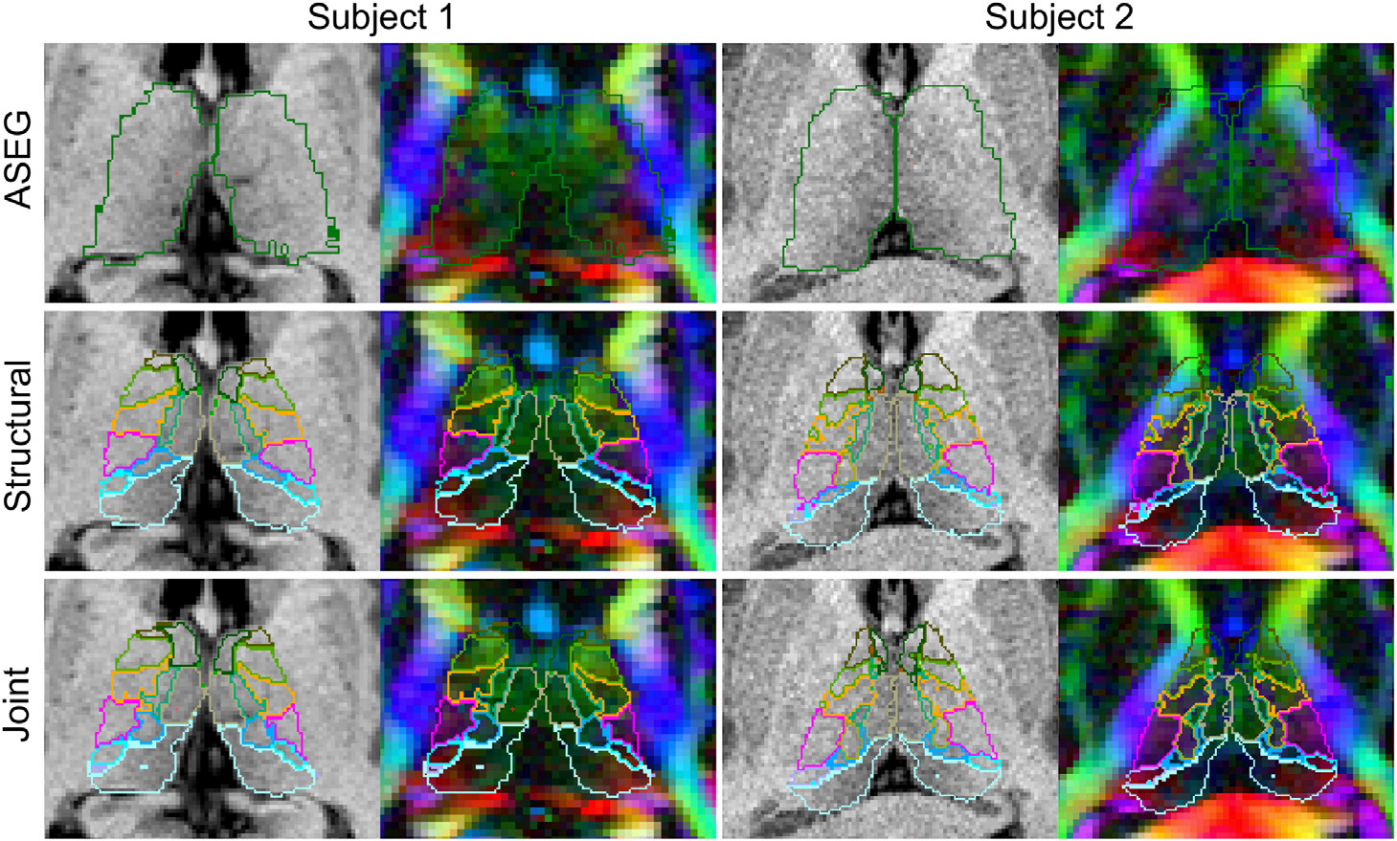
Comparison of representative axial slices from thalamic segmentations generated by FreeSurfer’s recon-all (*aseg.mgz*), structural and joint (DSW-beta) Bayesian segmentation on two HCP subjects. Coloured outlines correspond to the histological atlas labels listed in Table 1. These labels are grouped for further quantitative analysis. Comparisons of coronal and sagittal slices are shown in Section S.6. of the supplement.

Having individually tuned the mixture models and defined a manual protocol corresponding to our histological labels, the obvious next step is to assess the performance of our joint segmentation method on HCP quality data. A comparison of our joint segmentation to both the FreeSurfer whole thalamus segmentation (*aseg.mgz*) and our previous structural-only method are shown in Fig. 6. This figure shows each segmentation overlaid on both the T1-weighted sMRI and the DEC-FA for two healthy subjects.^1^

In both subjects the whole thalamus aseg segmentation, used as an initialisation for both Bayesian methods, shows obvious errors when overlaid on the DEC-FA, with more extreme over-segmentation for subject 2. In subject 1 the structural-only segmentation appears to compensate for these errors and provides an improved exterior boundary. However, our joint method shows marked improvement in the agreement of internal boundaries with colours displayed in the dMRI as well as a smaller improvement in the exterior boundary. This effect is much more pronounced in subject 2, where the initial over-segmentation of the thalamus propagates to the structural-only method but is corrected by the joint method.

Such observations provide compelling qualitative evidence for the efficacy of our new method. However, to fully evaluate its usefulness we must quantitatively assess both accuracy and repeatability.

#### 3.3.1 Direct evaluation with manual ground truth

**Fig. 7:**
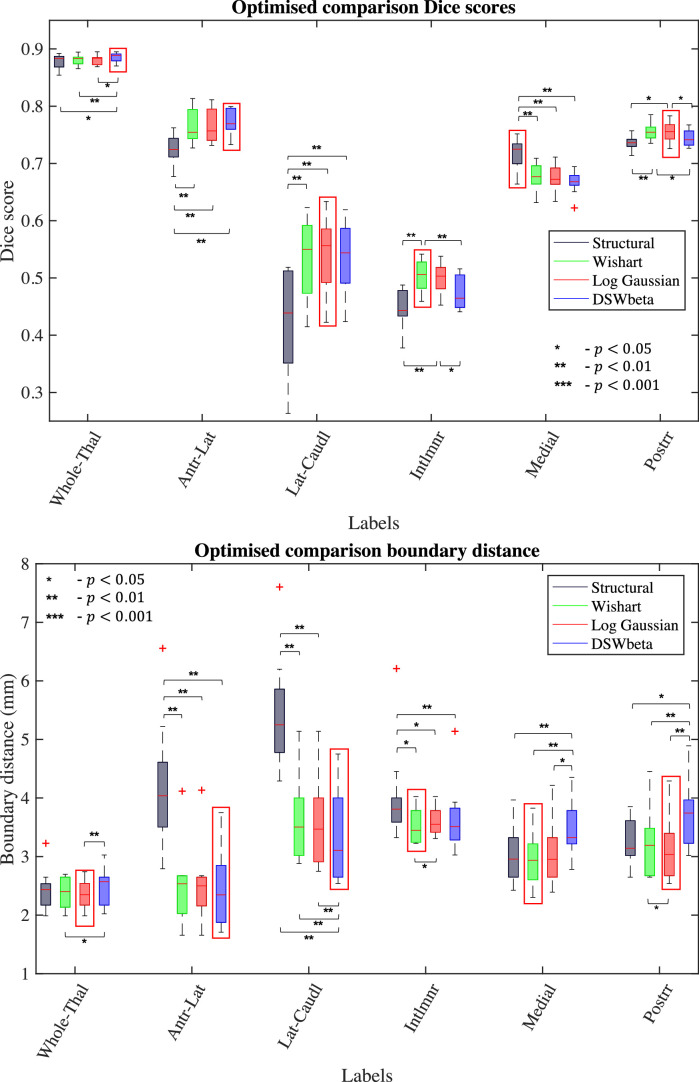
Dice score (top) and 95HD (bottom) comparison of automated thalamic segmentations to manual delineations of 10 HCP subjects. Scores are stated for our previous structural only method as well as the three likelihood implementations of our joint method. Asterisks denote significance level on Wilcoxon signed-rank test.

To provide a quantitative measure of segmentation quality, our anatomy expert (JA, assisted by MB) manually segmented images for 10 randomly selected subjects from the WashU-UMN HCP dataset (Van Essen et al., 2013) using criteria developed from the population template as described in Section 3.1. The manual segmentations were performed using a combination of T1-weighted and DEC-FA at a 1.25 mm isotropic resolution, corresponding to the native resolution of the diffusion data in HCP. We generated segmentations for these subjects using each of the three joint likelihood implementations from Section 2.3 as well as our previously published structural-only implementation (Iglesias et al., 2018). These automated segmentations, which have the resolution of the structural scans (0.7 mm), were resampled to 1.25 mm isotropic resolution and compared with the ground truth using DSC and 95HD. Dice scores and 95HD for the five groupings (in column one of Table 1) and the whole thalamus are shown in Fig. 7. We highlight the model achieving the best median value for each measurement as well as statistically significant differences between models (Wilcoxon signed-rank test).

All three joint segmentation methods show distinct improvements in both DSC and 95HD across multiple labels and smaller improvements in the whole thalamus exterior. Here, the structural-only, Wishart and Log-Gaussian implementations achieve median DSCs of 0.88 with a small increase to 0.89 for DSW-beta implementation. While this increase does achieve significance compared to the other three, it is countered by a small increase of 0.12 mm in median 95HD compared to the Wishart and log-Gaussian implementations. Even so, the 95HD for all methods was between 2.3 and 2.5 mm, equivalent to approximately 2 voxel widths on the manual segmentations.

A joint segmentation method obtained the best 95HD in each of the five label groups with particularly large improvements in the antero-lateral and lateral-caudal groups. Similarly, the joint methods outperform structural-only DSC in four of the five groups with lateral-caudal class showing an improvement of 10 Dice points. The only label class where the structural method outperforms the joint implementations is the medial class. This is expected as the medial-lateral contrast change is the only explicitly modelled interior boundary in the structural-only method. However, the 95HD measurement for the medial thalamus shows no significant differences between the structural implementation and the Wishart implementation, which performs best in this measurement.

There is comparatively little difference between the three diffusion likelihood implementations. The Wishart and Log-Gaussian implementations show the most similar results, while in the DSW-beta implementation small decreases in accuracy of the intralaminar and posterior classes are offset by improvements in the antero-lateral classes and whole thalamus exterior.

#### 3.3.2 Test-retest reliability analysis

In order to assess the test-retest reliability of the method (a crucial feature in large scale, multi-centre studies), we segmented images from 110 HCP subjects using two different sets of DTI images for each subject – one based on the b=1000 $s/mm^{2}$ shell and one based on the b=2000 $s/mm^{2}$ shell – and compared the outputs. While the results of such an experiment are optimistic when compared to experiments in which images are acquired with multiple scanners, it does enable thorough comparison within the same dataset; test-retest experiments with multiple acquisitions are described in Section 3.4.1 below.

**Fig. 8:**
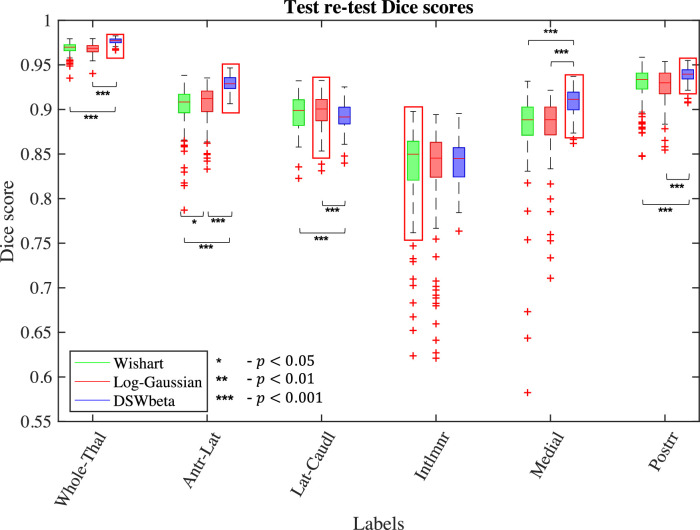
Dice score evaluation of test-retest reliability on 110 HCP subjects. For each subject, we performed two segmentations using DTI images obtained by fitting the tensor to the data from b=1000 $s/mm^{2}$ and b=2000 $s/mm^{2}$ shells separately and computed Dice scores for groups of labels in the two resulting segmentations. Asterisks denote significance level on Wilcoxon signed-rank test.

First we examine the effect of such an acquisition change on the groupings evaluated in Section 3.3.1. Dice scores for these groupings can be seen in Fig. 8. These results generally show that all three models are reasonably robust to such an acquisition change in HCP quality data, with a median Dice score of 0.85 or greater in each grouped label across all models and greater than 0.95 for the whole thalamus. However, the DSW-beta implementation does appear to be more robust. This model shows improved Dice scores with high significance in the whole thalamus, antero-lateral, medial and posterior groupings. Conversely, there is a slight but significant drop in the lateral-caudal grouping and there is no significant difference between the three models in the intralaminar grouping.

This increased stability of the DSW-beta implementation compared to the other models is also reflected in the individual label Dice scores. In the left-right averaged Dice scores for 25 labels we find that the DSW-beta achieves the highest median scores in 17 labels and differences from the winning model in a further 3 labels do not reach significance. Of the remaining 5 labels DSW-beta still achieves scores greater than 0.85 in the VPL and CM nuclei and greater than 0.75 for the MV(Re). The lowest Dice scores for all three methods are present in the VM, Pc and Pt nuclei. These are small nuclei, in the region of $2-5\;mm^{3}$, and consist of fewer than ten voxels in each hemisphere. Dice scores for individual nuclei from the DSW-beta implementation can be found in section S.7 of the supplement.

To account for these small classes, we also examine the volume measurements of each label. These volumes are calculated as the sum across voxels of the posterior probability of each label multiplied by the voxel size to account for voxels with multiple non-zero posteriors. Examining the intra-class correlation coefficients (*ICC*) for these volumes in the DSW-beta implementation shows the volumes are extremely stable between acquisition types. Looking at the left and right labels separately we find that 27 of the labels have ICCs above 0.9 with a further 20 having values above 0.8, indicating high correlation between the volume measurements generated by each acquisition type. In fact the ICCs for the remaining labels are also all above 0.75 apart from the right Pc with a value of 0.69, indicating that the volumes for the VM, Pc and Pt may still be used for volumetric analysis. The median label volumes and ICCs for the DSW-beta implementation can be found in section S.7 of the supplement.

### 3.4 Applications to conventional quality dMRI

While our method assumes that the resolution of the diffusion MRI approaches 1 mm isotropic (which is the case for many modern datasets, e.g., following the HCP protocol), it is of high interest to segment the thalamic nuclei in lower resolution scans for two reasons. First, because large amounts of legacy data were acquired at lower resolution. And second, because many current studies (e.g., ADNI, GENFI) still use those acquisitions, either in order not to deviate from the protocol used to acquire images earlier in the project or to accommodate acquisition constraints such as available scanner time. As explained in Section 2.3 above, compatibility with conventional quality data is actually the reason why we chose to model the diffusion tensor in our likelihood term, rather than using a more sophisticated, higher order model. Therefore, to assess our method on conventional quality scans, we perform both reliability analysis and indirect evaluation using two conventional quality datasets. In the first experiment we use a locally acquired dataset at the UCL DRC, which provides T1-weighted MPRAGEs and two dMRI scans for 21 healthy controls. In the second experiment we use both healthy controls and subjects with AD from the ADNI dataset.^2^

The resolution of the dMRI scans provided by these two datasets is heavily reduced from that of the HCP data. The voxels in the UCL DRC images encompass 8 times the volume of those in the HCP images, while the ADNI image voxels are 2.5 times larger than HCP, with double the slice thickness. This decrease in the resolution of such scans, compared to HCP, make manual delineation infeasible using the joint structural and DEC-FA criteria from Section 3.1. The large volumes of these voxels increase partial volume effects within the dMRI, obscuring boundaries, while the increased slice thickness makes it difficult to trace the first and last slices of every group. Instead, in Section 3.4.1 we perform test-retest reliability analysis and in Section 3.4.2 we perform indirect validation, using the ability to discriminate between subjects with AD and healthy controls as a proxy for segmentation accuracy.

#### 3.4.1 Test-retest reliability analysis

**Fig. 9:**
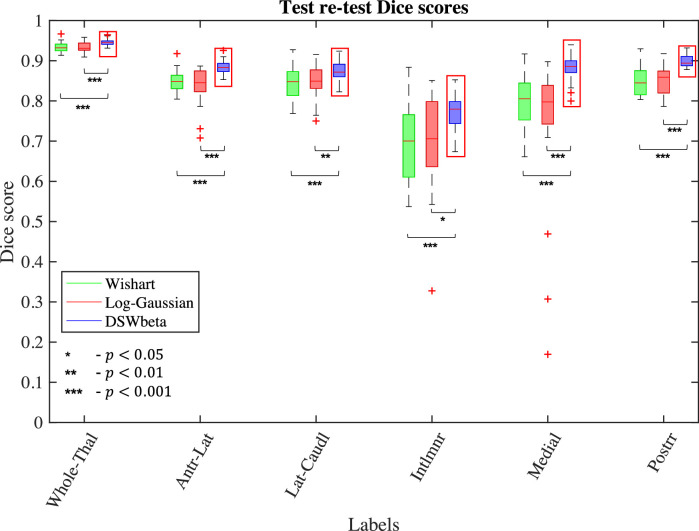
Dice score evaluation of test-retest reliability on conventional-quality data from 21 subjects acquired at the UCL Dementia Research Centre. For each subject, we performed two segmentations using dMRI data acquired in the same session using the same acquisition parameters and computed Dice scores for groups of labels. Asterisks denote significance level on Wilcoxon signed-rank test.

In order to assess the test-retest reliability of the method on lower resolution dMRI, we used a separate dataset, comprising 21 healthy volunteers (9 male, 12 female, aged 53 – 80 years) acquired at the UCL DRC. Three MRI sequences were performed for each subject in a single session: one T1-weighted MPRAGE 1.1 mm isotropic resolution; and two diffusion weighted acquisitions each consisting of 64 gradient directions at a b-value of 1,000 $s/mm^{2}$ and a 2.5 mm isotropic resolution. Using the two dMRI acquisitions as separate tests, segmentations were performed at a 1 mm isotropic resolution in the native orientation of the individual dMRI volumes before being resampled to the native space of the structural volume for calculation of test-retest Dice scores. Groupwise Dice scores for this experiment are shown in Fig. 9.

As expected from the increased voxel size and reduced quality of the data, the Dice scores in Fig. 9 are lower than those in Fig. 8, although median scores are still above 0.9 for whole thalamus and 0.8 for four of the five grouped labels. However, it is clearer from this plot that the DSW-beta implementation is the most robust to differences in dMRI, with the highest median Dice score in each category. This may be due the increased dimensionality of the Wishart and log-Gaussian models, meaning imprecise fitting of the tensor model caused by partial volume effects has a greater impact than for the more robust FA and principle direction model used by the DSW-beta likelihood.

As with the previous test-retest experiment, this increased stability of the DSW-beta is also reflected in the individual label Dice scores. In this case DSW-beta achieves the highest median scores in 21 labels and differences from the winning model in a further 2 labels do not reach significance when looking at left-right averaged Dice scores. Of the remaining labels, DSW-beta still achieves scores greater than 0.80 in the VLp, while the LD is a small nucleus in the region of 19 $mm^{3}$ and still achieves ICCs above 0.95 in both hemispheres. In fact, of all the nuclei, 20 show ICCs above 0.9, with a further 18 having values above 0.8 and all but 5 above 0.7, including for some small nuclei under $50\;mm^{3}$ where Dice scores are reduced. The median label volumes, Dice scores and ICCs for the DSW-beta implementation can be found in section S.7 of the supplement.

#### 3.4.2 Alzheimer’s disease study

So far we have performed experiments to evaluate both reliability and accuracy measures for the three joint models. While all three models show similar differences in accuracy compared to structural only segmentation on HCP quality data, generation of ground truth manual segmentations on conventional quality data was infeasible using the protocol from Section 3.1, due to the reduced resolution of the dMRI. To compensate for this we repeat an indirect evaluation experiment from our previous work (Iglesias, Insausti, Lerma-Usabiaga, Bocchetta, Van Leemput, et al., 2018, Iglesias, Van Leemput, Golland, Yendiki, 2019) in which we evaluate the utility of our segmentations in a scenario more closely resembling a classical group study.

Specifically, we examine the ability to discriminate between healthy controls and subjects with AD from the ADNI dataset using the volume measurements derived from the DSW-beta implementation as compared to the structural only and FreeSurfer whole thalamus segmentations. While the thalamus is less strongly affected in AD than other structures (e.g., the hippocampus), it is still expected to see bilateral atrophy of around 12%, with local shrinkage in the anterodorsal, centromedial, intralaminar and pulvinar nuclei (Pini et al., 2016). Despite this, volume measurements of whole thalamus segmentations can show poor discriminative ability, making improved discriminative ability from nuclei measures indicative of improved segmentation quality. The decision to focus on the DSW-beta implementation was taken due to the significantly improved reliability of the DSW-beta labels compared to both Wishart and log-Gaussian models in HCP quality and conventional quality scans, while accuracy on HCP quality scans remains comparable.

**Fig. 10:**
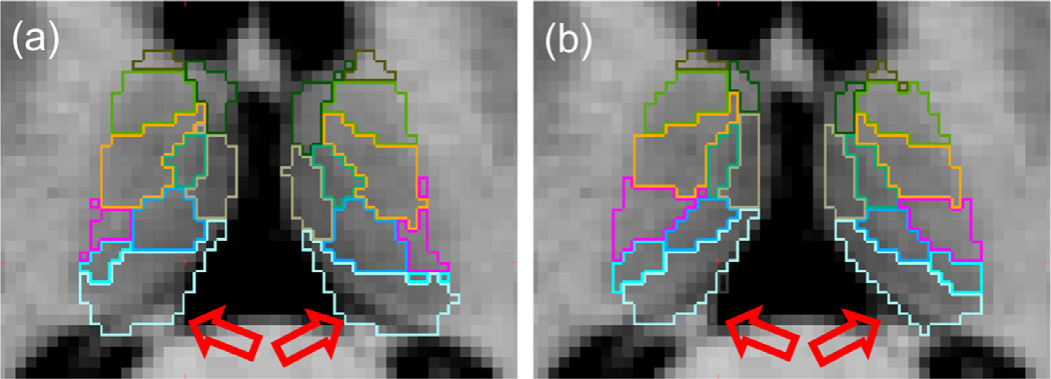
Comparison of thalamic segmentations of a subject from the ADNI dataset using equal (a) and reduced (b) dMRI likelihood weighting. Weighting the dMRI likelihood by the ratio of voxel volumes between sMRI and dMRI results in more accurate estimation of boundaries with heavy partial voluming in the diffusion channel, e.g., the CSF/posterior-thalamus boundary (red arrows). (For interpretation of the references to colour in this figure legend, the reader is referred to the web version of this article.)

First we consider 45 subjects with AD and 45 controls (73.7±18.0 years; 44 females total) from the ADNI. These subjects were initially processed for a study on connectivity differences in dementia (Frau-Pascual et al., 2019) and used for a classification experiment in our previous work (Iglesias et al., 2019). The data consisted of T1-weighted scans, with a resolution of 1.211 mm (sagittal), and dMRI with a resolution of 1.351.352.7 mm (axial). We fit the DTI model to the b=1000 $s/mm^{2}$ shell (41 directions), combined with 5 volumes at b=0. We then segmented each subject using the FreeSurfer recon-all stream as well as our previous structural only method and DSW-beta model joint implementation. However, initial examination of these subjects revealed some cases where the inclusion of the dMRI shifts boundaries in the segmentation due to the lower resolution of the dMRI data (and thus increased partial volume effects). An example is the over-segmentation of the thalamus into the CSF in Fig. 10a. We addressed this by allowing the contribution of the dMRI likelihood term to be reduced in proportion to the ratio between voxel volumes in the sMRI and dMRI volumes (Fig. 10b) as outlined in Section 2.5 and Section S.2 of the supplement.

**Fig. 11:**
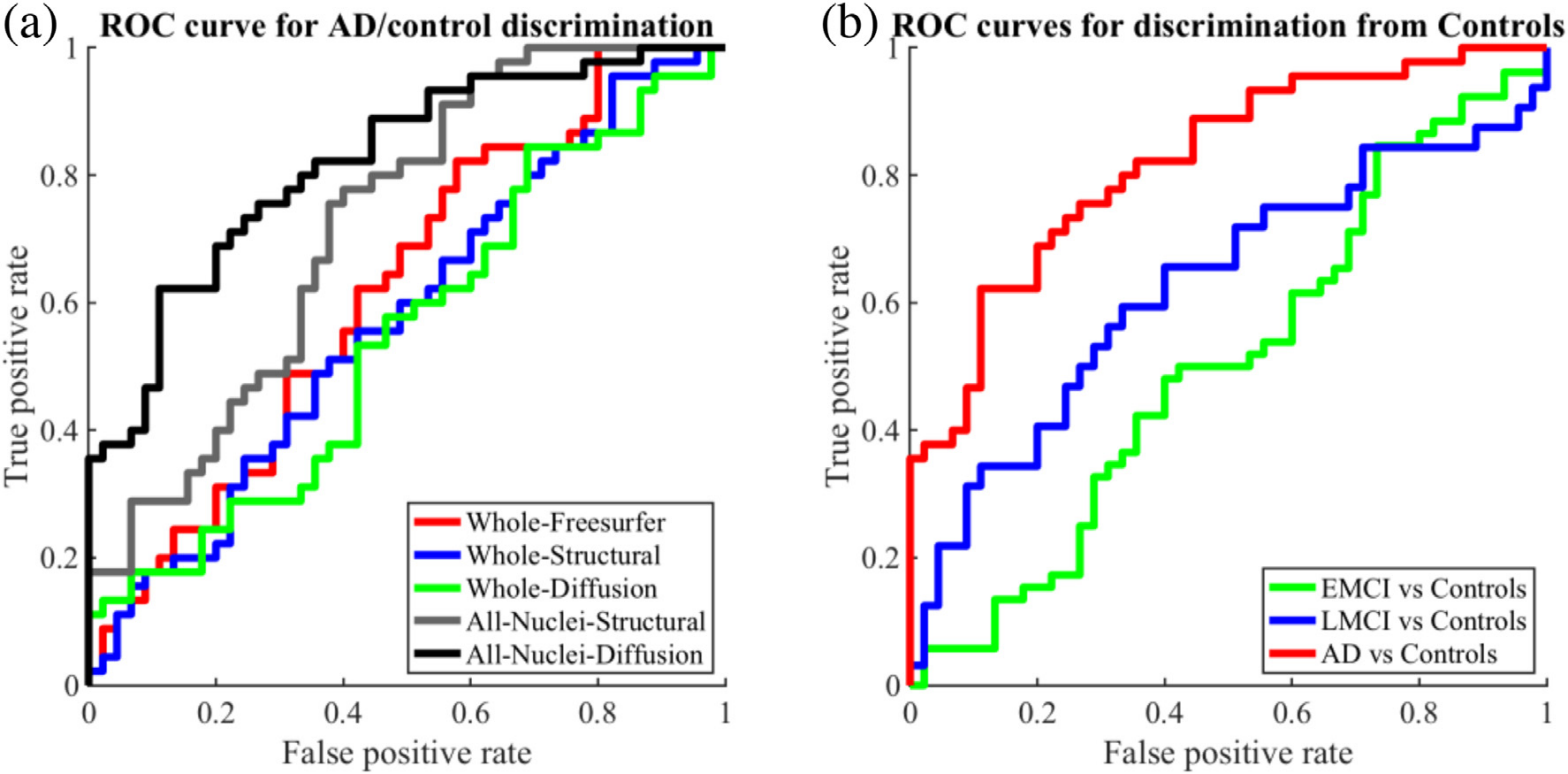
ROC curves for classification of subjects within the ADNI dataset based on thalamic volumes. a) Compares classification between AD and controls using 5 methods. b) Compares classification of subjects with AD, early and late MCI from healthy controls using the nuclei volumes from diffusion.

As in Iglesias et al. 2019, we computed receiver operating characteristic (*ROC*) curves for discrimination of subjects into the two classes using five approaches: three based on thresholding the volume of the whole thalamus (as given by the FreeSurfer recon-all stream, the structural segmentation, and the joint segmentation); and two based on thresholding the likelihood ratio given by a linear discriminant analysis (LDA, Fisher 1936) on the volumes of the histological nuclei (as given by the structural and joint segmentation). The resulting ROC curves are shown in Fig. 11(a) with the area under the curve (*AUC*), accuracy at the elbow and p-values for comparison of AUC values shown in Table 2.

**Table 2:** AUC, accuracy at elbow, and p-value for improved AUC values as given by a DeLong test.

	FreeSurfer	Structural	Diffusion
	(whole)	(nuclei)	(nuclei)
AUC	62.02%	72.30%	**81.98%**
Acc. at elbow	62.22%	68.89%	**75.56%**
p-value vs FreeSurfer		0.150	**0.004**
p-value vs Structural			**0.049**

From these curves we can see that all three methods relying on the total volume of the thalamus have poor discriminative ability, with little difference between using FreeSurfer, structural or joint segmentations. This contrasts to the nuclei specific methods, which both show marked improvements. Structural segmentation shows an increase of 10% AUC over FreeSufer’s whole thalamus and joint segmentation an increase of 20%. However, only the improvements of the joint method show statistical significance with p=.004 vs. FreeSurfer and p=.049 vs structural nuclei segmentation.

Tables 3 and 4 compare AUC values and Cohen’s d scores for the nuclei showing statistically significant differences between Alzheimer’s and controls (p<.05) in the joint and structural segmentation methods respectively. The most significant atrophy detected by the joint segmentation method was present in the medial portion of the PuM that was added to the atlas to model heterogeneity in the pulvinar. While the smaller sample size in the current study (N=90 vs N=374) resulted in lowered significance for some atrophy measurements and contributes to reduced AUC overall for the structural method compared to the experiment in (Iglesias et al., 2018), the medial PuM still reaches significance in joint segmentation after Bonferroni-correction for 26 multiple comparisons (p<.0019). Comparing these to the structural measurements, more structural labels reach significance at p<.05 but not after correction for multiple comparisons. The joint segmentation differentiates more between nuclei, while the structural volumes are more correlated, possibly due to the two component model used in the structural likelihood model. We note that unlike our previous work the LGN and MGN do not contribute significantly to atrophy in either method, this is likely due to modification of these labels in the latest version of the atlas available in FreeSurfer 7.2.

**Table 3:** Thalamic nuclei showing statistically significant differences between Alzheimer’s and controls for the joint segmentations, sorted by increasing p-value (Wilcoxon rank-sum).

Structure	AUC	Cohen’s d	p-value
PuM-medial	71.60%	0.7850	0.0004
MDm	66.77%	0.5827	0.0062
MDl	62.96%	0.3005	0.0345

**Table 4:** Thalamic nuclei showing statistically significant differences between Alzheimer’s and controls for the structural segmentations, sorted by increasing p-value (Wilcoxon rank-sum).

Structure	AUC	Cohen’s d	p-value
MDm	68.20%	0.7478	0.0030
MDl	68.05%	0.4868	0.0032
AV	67.31%	0.5432	0.0047
VA	66.12%	0.5626	0.0085
PuA	63.85%	0.4631	0.0239

Given the improved discriminative ability of the jointly segmented nuclei for AD vs control’s, we applied the DSW-beta segmentation method to 84 additional subjects from ADNI. These consisted of 52 subjects (73.7Ø18.5, 11 females) with early mild cognitive impairment (*EMCI*) and 32 subjects (73.2Ø16.7, 19 females) with late mild cognitive impairment (*LMCI*). The corresponding ROC curves for discrimination between these groups and controls in Fig. 11(b) show a smooth, progressive transition across the four stages of the disease. This highlights the ability of our method to pick up on more subtle volume differences from LMCI (AUC 62.57%, Acc. at elbow 66.23%) although not from EMCI (AUC 50.56%, Acc. at elbow 57.73%).

## 4 Discussion and conclusion

In this article, we have presented and tested a novel segmentation method for thalamic subregions from structural and diffusion MRI. Building on the Bayesian segmentation literature, we propose an algorithm to incorporate likelihood models of both structural and diffusion MRI into a single *joint* segmentation. By combining this with novel likelihood models of dMRI, we obtain accurate identification of the main thalamic regions. Through this method the information in structural MRI enables placement of boundaries in regions with strong contrast (e.g. the medial boundary with the ventricles) with high precision, attributed to its higher resolution; the diffusion information enables the accurate segmentation of boundaries that are invisible in typical structural MRI sequences. Furthermore, we have presented an improved version of our previous histological atlas, which enables more accurate modelling of diffusion MRI in the cerebral white matter. The proposed method will be distributed with FreeSurfer and is widely applicable because the likelihood: *(i)* relies on a simple DTI model, which makes it compatible with virtually every diffusion dataset; *(ii)* adjusts to different resolutions by correcting for voxel sizes; and *(iii)* relies on an unsupervised model that is robust against changes in MR contrast.

We have conducted extensive experiments with manual segmentations, test-retest acquisition, and group studies – including datasets with different resolutions. The results have shown that the joint model exploiting the diffusion information improves accuracy over structural-only segmentation. Moreover, we have also found that the varying resolution gap between structural and diffusion MRI may be accommodated by weighting the diffusion likelihood term to account for voxel size differences, thus bypassing the need to explicitly model partial voluming – which quickly becomes intractable, particularly in multi-modal images defined on different voxel grids. While both our proposed likelihood model (DSW-beta) and the two competing alternatives showed similar levels of improved accuracy over structural-only segmentation when compared with manual delineations, we found the DSW-beta distribution to have the highest test-retest reliability and to be the most robust at lowered dMRI resolution.

Our proposed method has a large number of design choices, particularly linked to the specification of shared parameters across classes in the structural and diffusion mixture models. We set these parameters with the combination of expert prior knowledge, a labelled template, and a well-known approach from the decision making literature (TOPSIS). While this approach is suboptimal (our prior knowledge is imperfect; a single template is biased towards a certain population, contrast, and resolution; and TOPSIS’s criteria may not necessarily be ideal), it yielded groupings that worked well in practice for different datasets with different resolution.

This work has a number of limitations. In particular, there are aspects of our modelling which could be further improved, or which require additional investigation. For example, we do not explicitly model the partial volume effect; while accounting for the voxel size ratio mitigated this problem in our experiments, it is possible that it does not suffice for more extreme ratios. This could be addressed with further experimentation on datasets with varying dMRI resolution or solutions based on CNNs.

Another modelling decision that could be investigated further is the reflective symmetry constraint we impose on dMRI distributions for contralateral structures. Our approach attempts to protect against abnormal structural asymmetry by deriving the plane of reflection from the reflected dMRI likelihood distributions rather than anatomical markers. We expect that asymmetries uniformly affecting a hemisphere would cause the estimated reflective plane to be rotated from the midline, but that segmentation accuracy would remain unaffected. More focal pathologies that cause asymmetrical directionality are likely to result in less heavily peaked likelihood distributions for the affected labels, equivalent to an increased variance for a Gaussian model. This could potentially impact segmentation accuracy for affected contralateral structures, though the impact is expected to be mitigated by the contribution of the prior and structural likelihoods, and their contribution to the reflection objective would be reduced limiting their effect on other labels. Pathologies with a larger impact on brain anatomy, such as lesions and tumours, are likely to affect segmentation accuracy for the additional reason that they are not explicitly modelled by our atlas, as is the case with many methods. Determining the effect of such asymmetries, and testing the performance of our methods with and without reflection, require further work and validation, so that the method can be reliably applied to a wider range of conditions.

There are also opportunities to improve the validation of our method, e.g. by assessing the quality of the manual labels through intra- and inter-rater variability or investigating other methods to generate ground truth segmentations. We designed our manual segmentation protocol to allow comparison of regions discernible from a combination of 3T T1-weighted MPRAGE images and HCP quality DEC-FA. This resulted in the segmentation of ten thalamic regions, which were further combined into five groupings for evaluation, limiting the detail of our ground truth comparisons. Improved accuracy for such groups of nuclei is a positive step towards validation of the separate labels, and registration of grouped boundaries in a hierarchical approach has been shown to improve segmentation accuracy (Liu et al., 2020). However, full validation of our nuclei level labels remains to be done and will require datasets that pair standard sMRI/dMRI with advanced imaging in which nuclei level structures are manually identifiable.

Advanced 7T MRI sequences can show improved contrast for thalamic nuclei, with manual segmentations having been generated from both white-matter-nulled (WMn) MPRAGE sequences (Su et al., 2019) and susceptibility weighted imaging (Liu et al., 2020) to validate thalamic segmentation algorithms. For example, Liu et al. (2020) demonstrate Dice scores of between 0.53 (habenula) and 0.9 (whole pulvinar) when applying their semi-automated method to 3T T1-weighted images for which an accurate exterior thalamic boundary has been provided as an input. Similarly Su et al. (2019) demonstrate Dices scores between 0.64 (ventral lateral anterior) and 0.89 (mediodorsal) using multi-atlas segmentation on their 7T WMn-MPRAGE images. Additionally, while dMRI clustering methods (Battistella et al., 2017) have shown limited qualitative alignment to histological labellings, advanced dMRI in the form of short-track tract density imaging has been used to manually identify 13 histologically guided nuclei (Basile et al., 2021). Currently there are no standard guidelines when it comes to neuroimaging of the thalamus; harmonisation of competing thalamic label definitions is a focus in the thalamic segmentation community, with ongoing efforts from the international ThAlamic nuclei Neuroimaging GrOup (TANGO), mirroring a similar effort for hippocampal subfields (Wisse et al., 2017).

The presented method will be publicly available in FreeSurfer as an extension of our current structural-only code. As high-resolution diffusion data become increasingly accessible, algorithms that can exploit them to produce accurate segmentations – particularly for boundaries that are invisible in structural MRI – have the potential to greatly enhance neuroimaging studies.

## Acknowledgments

This work was primarily funded by Alzheimers Research UK (ARUK-IRG2019A003). PGs work in this area was supported by NIH NIBIB NAC P41EB015902 AYs work in this area was supported by NIH grants R01 EB021265 and R56 MH121426. DCAs work in this area was supported by EPSRC grant EP/R006032/1 and Wellcome Trust award 221915/Z/20/Z. The Dementia Research Centre is supported by Alzheimer’s Research UK, Alzheimer’s Society, Brain Research UK, and The Wolfson Foundation. This work was supported by the National Institute for Health Research (NIHR) Queen Square Dementia Biomedical Research Unit and the University College London Hospitals Biomedical Research Centre, the Leonard Wolfson Experimental Neurology Centre (LWENC) Clinical Research Facility, and the UK Dementia Research Institute, which receives its funding from UK DRI Ltd, funded by 10.13039/100007249the UK Medical Research Council, Alzheimer’s Society and Alzheimer’s Research UK. This project has received funding from the European Unions Horizon 2020 research and innovation program under the Marie Sklodowska-Curie grant agreement No. 765148, as well as from the National Institutes Of Health under project number R01NS112161. MB is supported by a Fellowship award from the Alzheimers Society, UK (AS-JF-19a-004-517). MBs work was also supported by the UK Dementia Research Institute which receives its funding from DRI Ltd, funded by 10.13039/100007249the UK Medical Research Council, Alzheimers Society and Alzheimers Research UK. JDR is supported by the Miriam Marks Brain Research UK Senior Fellowship and has received funding from an MRC Clinician Scientist Fellowship (MR/M008525/1) and the NIHR Rare Disease Translational Research Collaboration (BRC149/NS/MH). JEI is supported by the European Research Council (Starting Grant 677697, project BUNGEE-TOOLS) and the NIH (1RF1MH123195-01 and 1R01AG070988-01).

The collection and sharing of the ADNI data was funded by the 10.13039/100007333Alzheimer’s Disease Neuroimaging Initiative (National Institutes of Health Grant U01 AG024904) and Department of Defence (W81XWH-12-2-0012). ADNI is funded by the 10.13039/100000049National Institute on Aging, the National Institute of Biomedical Imaging and Bioengineering, and the following: Alzheimer’s Association; Alzheimer’s Drug Discovery Foundation; BioClinica, Inc.; Biogen Idec Inc.; Bristol-Myers Squibb Company; Eisai Inc.; Elan Pharmaceuticals, Inc.; Eli Lilly and Company; F. Hoffmann-La Roche Ltd and affiliated company Genentech, Inc.; GE Healthcare; Innogenetics, N.V.; IXICO Ltd.; Janssen Alzheimer Immunotherapy Research & Development, LLC.; Johnson & Johnson Pharmaceutical Research & Development LLC.; Medpace, Inc.; Merck & Co., Inc.; Meso Scale Diagnostics, LLC.; NeuroRx Research; Novartis Pharmaceuticals Corporation; Pfizer Inc.; Piramal Imaging; Servier; Synarc Inc.; and Takeda Pharmaceutical Company. The Canadian Institutes of Health Research is providing funds for ADNI clinical sites in Canada. Private sector contributions are facilitated by the Foundation for the National Institutes of Health. The grantee is the Northern California Institute for Research and Education, and the study is coordinated by the Alzheimer’s Disease Cooperative Study at the University of California, San Diego. ADNI is disseminated by the Laboratory for Neuro Imaging at the University of Southern California.

## CRediT authorship contribution statement

**Henry F.J. Tregidgo:** Methodology, Software, Formal analysis, Writing – original draft. **Sonja Soskic:** Validation, Writing – review & editing. **Juri Althonayan:** Investigation. **Chiara Maffei:** Data curation, Resources, Writing – review & editing. **Koen Van Leemput:** Software. **Polina Golland:** Conceptualization, Writing – review & editing. **Ricardo Insausti:** Investigation. **Garikoitz Lerma-Usabiaga:** Investigation. **César Caballero-Gaudes:** Investigation. **Pedro M. Paz-Alonso:** Investigation, Writing – review & editing. **Anastasia Yendiki:** Conceptualization, Resources, Writing – review & editing. **Daniel C. Alexander:** Conceptualization, Writing – review & editing. **Martina Bocchetta:** Conceptualization, Investigation, Writing – review & editing. **Jonathan D. Rohrer:** Conceptualization, Funding acquisition. **Juan Eugenio Iglesias:** Conceptualization, Methodology, Software, Funding acquisition.

## Declaration of Competing Interest

The authors declare that they have no known competing financial interests or personal relationships that could have appeared to influence the work reported in this paper.

## Data Availability

The probabilistic atlas and segmentation tool will be made publicly available as part of the neuroimaging package FreeSurfer (https://freesurfer.net/fswiki/ThalamicNucleiDTI). Publicly available raw data used in the evaluation of this method may be obtained from the Human Connectome Project (http://www.humanconnectomeproject.org/) and the Alzheimer’s Disease Neuroimaging Initiative (https://adni.loni.usc.edu/). The UCL dataset that support the findings of this study are not publicly available due to ethical restrictions.

## Appendix A Supplementary materials

Supplementary Data S1 Supplementary Raw Research Data. This is open data under the CC BY license http://creativecommons.org/licenses/by/4.0/ Supplementary Data S1

Supplementary Data S2 Supplementary Raw Research Data. This is open data under the CC BY license http://creativecommons.org/licenses/by/4.0/ Supplementary Data S2

## Footnotes

1 The joint segmentation shown here uses our DSW-beta likelihood model and the structural method has been optimised for the HCP dataset by tuning of the stiffness parameter. For a visual comparison of all likelihood models and the default structural segmentation please see the Section S.6 of the supplement.

2 The ADNI was launched in 2003 by the National Institute on Ageing, the National Institute of Biomedical Imaging and Bioengineering, the Food and Drug Administration, private pharmaceutical companies and non-profit organisations, as a $60 million, 5-year public-private partnership. The main goal of ADNI is to test whether MRI, positron emission tomography (PET), other biological markers, and clinical and neuropsychological assessment can be combined to analyse the progression of MCI and early AD. Markers of early AD progression can aid researchers and clinicians to develop new treatments and monitor their effectiveness, as well as decrease the time and cost of clinical trials. The Principal Investigator of this initiative is Michael W. Weiner, MD, VA Medical Center and University of California San Francisco. ADNI is a joint effort by co-investigators from industry and academia. Subjects have been recruited from over 50 sites across the U.S. and Canada. The initial goal of ADNI was to recruit 800 subjects but ADNI has been followed by ADNI-GO and ADNI-2. These three protocols have recruited over 1500 adults (ages 5590) to participate in the study, consisting of cognitively normal older individuals, people with early or late MCI, and people with early AD. The follow up duration of each group is specified in the corresponding protocols for ADNI-1, ADNI-2 and ADNI-GO. Subjects originally recruited for ADNI-1 and ADNI-GO had the option to be followed in ADNI-2. For up-to-date information, see http://www.adni-info.org.
